# High-parameter cytometry unmasks microglial cell spatio-temporal response kinetics in severe neuroinflammatory disease

**DOI:** 10.1186/s12974-021-02214-y

**Published:** 2021-07-26

**Authors:** Alanna G. Spiteri, Rachel L. Terry, Claire L. Wishart, Thomas M. Ashhurst, Iain L. Campbell, Markus J. Hofer, Nicholas J. C. King

**Affiliations:** 1 Discipline of Pathology, Faculty of Medicine and Health, School of Medical Sciences, The University of Sydney, Sydney, Australia; 2 Charles Perkins Centre, The University of Sydney, Sydney, Australia; 3 Current Address: Children’s Cancer Institute, Randwick, New South Wales Australia; 4 Current Affiliation: Cancer Immunology Program, Peter MacCallum Cancer Centre, Melbourne, Victoria Australia; 5 Sydney Cytometry Facility, The University of Sydney and Centenary Institute, Sydney, Australia; 6 Ramaciotti Facility for Human Systems Biology, The University of Sydney and Centenary Institute, Sydney, Australia; 7 Marie Bashir Institute for Infectious Diseases and Biosecurity (MBI), Faculty of Medicine and Health, Sydney Medical School, The University of Sydney, Sydney, Australia; 8 School of Life and Environmental Sciences, The University of Sydney, Sydney, Australia; 9 Nano Institute, The University of Sydney, Sydney, Australia

**Keywords:** Microglia, Neuroinflammation, Viral encephalitis, High-dimensional cytometry, Flavivirus, Immune-mediated pathology

## Abstract

**Background:**

Differentiating infiltrating myeloid cells from resident microglia in neuroinflammatory disease is challenging, because bone marrow-derived inflammatory monocytes infiltrating the inflamed brain adopt a ‘microglia-like’ phenotype. This precludes the accurate identification of either cell type without genetic manipulation, which is important to understand their temporal contribution to disease and inform effective intervention in its pathogenesis. During West Nile virus (WNV) encephalitis, widespread neuronal infection drives substantial CNS infiltration of inflammatory monocytes, causing severe immunopathology and/or death, but the role of microglia in this remains unclear.

**Methods:**

Using high-parameter cytometry and dimensionality-reduction, we devised a simple, novel gating strategy to identify microglia and infiltrating myeloid cells during WNV-infection. Validating our strategy, we (1) blocked the entry of infiltrating myeloid populations from peripheral blood using monoclonal blocking antibodies, (2) adoptively transferred BM-derived monocytes and tracked their phenotypic changes after infiltration and (3) labelled peripheral leukocytes that infiltrate into the brain with an intravenous dye. We demonstrated that myeloid immigrants populated only the identified macrophage gates, while PLX5622 depletion reduced all 4 subsets defined by the microglial gates.

**Results:**

Using this gating approach, we identified four consistent microglia subsets in the homeostatic and WNV-infected brain. These were P2RY12^hi^ CD86^−^, P2RY12^hi^ CD86^+^ and P2RY12^lo^ CD86^−^ P2RY12^lo^ CD86^+^. During infection, 2 further populations were identified as 'inflammatory' and 'microglia-like' macrophages, recruited from the bone marrow. Detailed kinetic analysis showed significant increases in the proportions of both P2RY12^lo^ microglia subsets in all anatomical areas, largely at the expense of the P2RY12^hi^ CD86^−^ subset, with the latter undergoing compensatory proliferation, suggesting replenishment of, and differentiation from this subset in response to infection. Microglia altered their morphology early in infection, with all cells adopting temporal and regional disease-specific phenotypes. Late in disease, microglia produced IL-12, downregulated CX3CR1, F4/80 and TMEM119 and underwent apoptosis. Infiltrating macrophages expressed both TMEM119 and P2RY12 de novo, with the microglia-like subset notably exhibiting the highest proportional myeloid population death.

**Conclusions:**

Our approach enables detailed kinetic analysis of resident vs infiltrating myeloid cells in a wide range of neuroinflammatory models without non-physiological manipulation. This will more clearly inform potential therapeutic approaches that specifically modulate these cells.

**Supplementary Information:**

The online version contains supplementary material available at 10.1186/s12974-021-02214-y.

## Introduction

Neurotropic virus infection generates an inflammatory milieu in the central nervous system (CNS) in which innate myeloid lineage cells, in particular, monocyte-derived macrophages (MDM), are principal responders. MDMs originate as monocytes in the bone marrow (BM), which is both a factory and reservoir for these cells, replenishing the blood and various tissues over time. Over the course of infection in the mouse, Ly6C^hi^ inflammatory monocytes leave the BM in increasing numbers, migrating via the blood to the CNS in response to CCL2 produced by infected neurons [1]. These cells infiltrate the CNS and contribute to neuronal damage, seizures and mortality [1–3]. Notably, interruption of monocyte immigration into the brain using antibody blockade against VLA-4 or administration of immune-modifying particles dramatically reduces immigration of these cells into the CNS, resulting in long-term survival, with robust immunity to rechallenge [1–4]. While the role of MDMs in immune-mediated pathology in response to an infectious existential threat has been well characterised, the contribution of the resident myeloid cells in the CNS parenchyma has not.

Microglia are CNS-resident macrophages that seed the CNS from the yolk sac as KIT^+^ erythromyeloid precursors [5, 6] and renew in situ over a lifetime [7–9]. Understanding and tracking the specific roles of microglia and MDMs in neuroinflammation has until recently been substantially hindered by the lack of experimental and analytical systems capable of clearly discriminating between these cell types. Historically, the overlapping expression of identifying surface markers required adoptive transfers, parabiosis, bone marrow reconstitutions or chimeric animals to resolve populations, all of which create non-physiological conditions that may confound interpretation [10]. However, ‘microglia-specific markers’, and agents that deplete microglia, have begun to enable dissection of their roles in CNS pathology. Moreover, the emerging understanding of the complexity of the microenvironment in informing the phenotype of both cell types [11–14], has shifted the focus from in vitro to in vivo investigation of these cells, elucidating and emphasising their interdependent functions during disease.

Neuroinflammation is characteristic of many CNS pathologies, including autoimmunity, stroke, neurodegeneration and encephalitis. However, in models of multiple sclerosis (i.e.*,* experimental autoimmune encephalomyelitis (EAE)), ischemia, Alzheimer’s disease (AD) and amyotrophic lateral sclerosis (ALS), the inflammatory insult and infiltrate tends to be focal and is less severe than that seen in viral encephalitis induced, for example, by West Nile virus (WNV) infection. WNV encephalitis (WNE) is associated with wide-spread neuronal infection and substantial infiltration of MDMs, which ultimately drive mortality [1, 4]. This makes the clear identification of microglia particularly challenging.

WNV is a mosquito-borne positive-stranded neurotropic RNA virus that first came to western attention in New York in 1999 [15] and is now the most widely distributed flavivirus worldwide [16, 17]. While about 80% of human infection is asymptomatic, neuroinvasion, which occurs in ~1% of cases, carries a 10% fatality rate [18–20]. Elderly and immunocompromised individuals are at increased risk of neuroinvasive disease [21, 22]. No vaccine has yet been licenced for humans and although early supportive treatment can improve clinical outcomes [20], the precise mechanisms of disease that could inform efficacious therapeutic intervention have yet to be elucidated. One of the unresolved questions surrounding the influx of myeloid cells in the CNS during this disease, is how their role differs from the resident microglia.

The development of PLX5622, a small molecule colony stimulating factor 1 receptor (CSF1R) inhibitor, which crosses the blood-brain barrier (BBB) and depletes microglia [23], has recently prompted experiments in a range of virus encephalitis models. These studies point to a neuroprotective role for microglia early in disease. Thus, treatment with PLX5622 in mice infected with West Nile virus (WNV), Japanese encephalitis virus (JEV), Theiler’s encephalomyelitis virus (TMEV), pseudorabies virus (PRV) and murine hepatitis virus (MHV) resulted in enhanced virus titres [24] and mortality [25–29]. More recently, it has been shown that PLX5622 affects peripheral lymphoid and myeloid compartments [30], arguing for caution when interpreting data where PLX5622 has been assumed to be microglia-specific. In vesicular stomatitis virus (VSV) infection, alternative microglial depletion methods produced similar findings [31, 32].

Some of these studies implicate a role for microglia in effective T cell responses [26, 27, 29], cross-presentation to cytotoxic T cells [32], monocyte recruitment and maturation [24, 27, 29] and phagocytosis of infected neurons [24]. On the other hand, microglia have been implicated in hippocampus-dependent learning and memory deficits after recovery from neuroinvasive Zika and WNV infection [33, 34]. However, to our knowledge, no studies have attempted to identify and immunophenotype microglia or examine their behaviour in any detail in the intact brain over the course of lethal viral encephalitic infection.

We report a novel flow cytometric gating strategy to identify and characterise infiltrating macrophages and microglia in homoeostasis and severe neuroinflammation. During WNE, infiltrating macrophage populations expressed standard myeloid and even ‘microglia-specific’ markers. In contrast, four distinct microglial phenotypes, distinguishable by their CD86 and P2RY12 expression, were found in both homeostasis and disease. Microglia proliferated early in infection, but by day 7 had decreased in number, significantly altering their phenotypic, morphological and cytokine status. Further immunophenotypic analysis showed that microglia and macrophages exhibit spatial and temporal disease-specific phenotypes during infection.

## Materials and methods

### Mice

Female 9-10-week-old C57BL/6 mice were obtained from the Animal Resource Centre (ARC) (Western Australia, Australia) and kept in individually ventilated cages under specific pathogen-free conditions with access to food and water ad libitum—in accordance with National Health and Medical Research Council’s ethical guidelines. All experiments were completed with animal ethics approval number K20/05-2016/976 approved by the University of Sydney Animal Ethics Committee.

### WNV infection

Mice were anesthetised before they were intranasally infected with 1.2 × 10^5^ plaque forming units (PFU) of WNV delivered in 10 μL (a dose of WNV that is lethal in 100% of mice (lethal dose 100%, LD_100_)) of sterile PBS (as previously described [1]). The original stock acquired from The John Curtin School of Medical Research (ACT, Australia) was propagated alternately in C57BL/6 suckling mouse brains and in vitro in Vero cells [3]. Mice were sacrificed no later than day post-infection (dpi) 7.

### PLX5622 treatment

Plexxikon Inc. (USA) provided the PLX5622 which was formulated in AIN-76A standard chow by Research diets (USA). Mice were fed PLX5622 for 14 days.

### Intraperitoneal delivery of blocking antibodies

Monoclonal blockade antibodies, anti-CCL2 (BE0185), anti-VLA-4 (BE0071) and anti-Ly6C (BE0203) (BioXcell, USA) (all 200 μg of each antibody in 250 μL total volume), were prepared in sterile PBS and injected interperitoneally at dpi 5 and 6.

### Intravenous delivery of PKH67

As per the manufacturer’s instructions, PKH67 cell-linker was mixed with diluent C (Sigma-Aldrich, MO, USA) prior to use. PKH67 cell-linker was used at a tenfold higher concentration than recommended for in vitro cell staining and injected intravenously. Dye was injected 3 h prior to tissue collection to stain recently infiltrating cells and avoid staining resident parenchymal brain cells with the sporadic breakdown of the BBB.

### Intraperitoneal delivery of BrdU

Mice were injected intraperitoneally with 1 mg of BrdU (Sigma-Aldrich, MO, USA) in 200 μL sterile PBS 3 h before sacrifice.

### Flow cytometry

**Fig. 1:**
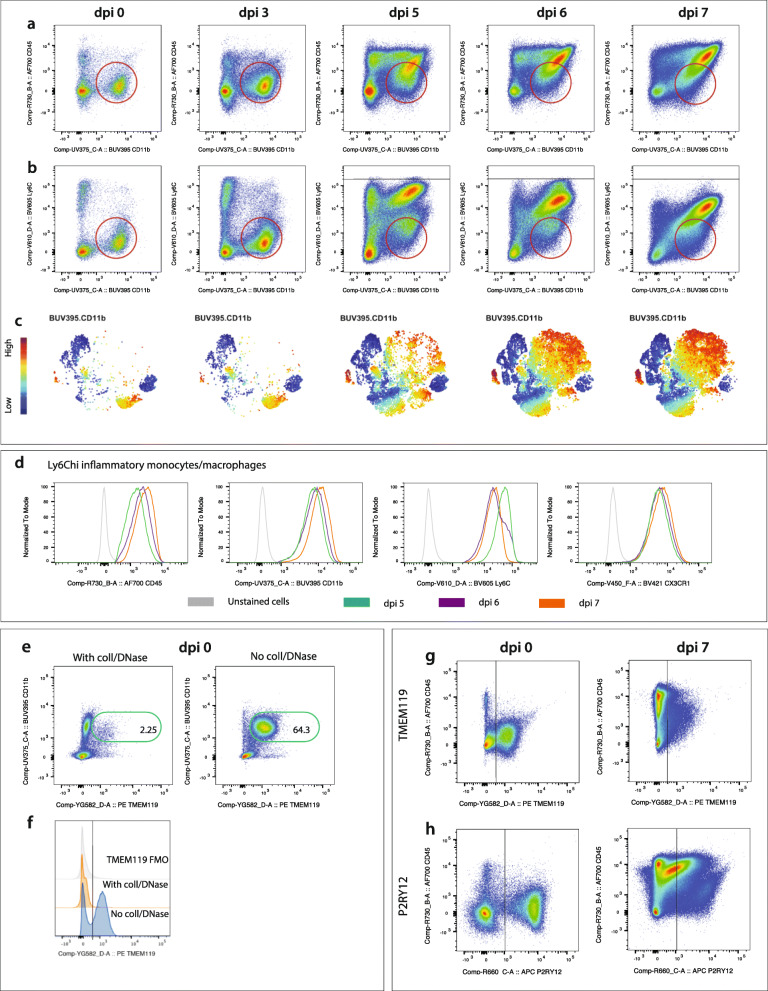
Microglia cannot be reliably distinguished by standard macrophage or microglia-specific markers during severe inflammation. **a**, **b** FACs plots showing the expression of CD45 vs CD11b (**a**) and Ly6C vs CD11b (**b**) on live, Ly6G^−^ cells from murine brains at day 0, 3, 5, 6 and 7 post WNV-infection. Red circles show microglia in dpi 0 and 3 brains, and a mixed population of microglia and macrophages in dpi 5-7 brains. **c** tSNE plots showing the infiltration and accumulation of Ly6C^hi^ cells from dpi 5-7. **d** Histograms showing the downregulation of Ly6C, and upregulation of CD45, CD11b and CX3CR1 on Ly6C^hi^ macrophages from dpi 5-7. **e** FACs plots showing the expression of ‘microglia-specific’ marker TMEM119 on live, Ly6G^−^ cells in mock-infected murine brains digested with and without collagenase and DNase. Values on FACs plots represent the frequency of live, Ly6G^−^, TMEM119^+^ cells. **f** Histogram showing the lack of expression of TMEM119 on live, Ly6G^−^ cells from mock-infected brains processed with collagenase and DNase. Fluorescence minus one (FMO) for TMEM119 is shown in grey. **g-h** FACs plots showing the expression of ‘microglia-specific’ markers TMEM119 (**g**) and P2RY12 (**h**) on live, Ly6G^−^ cells at WNV dpi 0 and 7. All data shown is representative of at least two independent experiments

Mice were anaesthetised and perfused transcardially with sterile PBS before tissue was collected. To avoid batch effects between timepoints, animals were infected on separate days in a biosafety cabinet (controlled environment) so tissue could be processed, stained and acquired together. Thus, in any instance where fluorescence intensity is compared (i.e.*,* in tSNE’s, heatmaps or histograms), samples were processed in a single batch with the same antibody panel. Bone marrow and brains were collected in ice cold PBS, while blood was collected in 50 mM EDTA. Brains were processed into single cell suspensions in PBS and DNase I (DN25, 0.05 mg/mL) and collagenase (5 mg/mL) (Sigma-Aldrich, MO, USA) using the gentleMACS dissociator (Miltenyi Biotec, Bergisch Gladbach, Germany). In some experiments, enzyme digestion was omitted to detect TMEM119 expression (Figs. 1e, 4, 5g-i and 6). Subsequently, a 30%/80% Percoll gradient was used to isolate the cells from brain homogenates. After tissue processing, live cells were counted with trypan blue (0.4%) on a haemocytometer. Single cell suspensions were incubated with purified anti-CD16/32 and UV-excitable LIVE/DEAD (UVLD) Blue (Invitrogen) or Zombie Aqua (Biolegend) and subsequently stained with a cocktail of fluorescently-labelled antibodies. Multiple panels were used to accommodate the incorporation of different dyes, including DAF-FM, PKH67, CFSE, as well as to ensure BrdU, annexin and cytokines (IL-12/IL-23 p40 and IFN-γ) were on bright fluorophores. Approximately, 8 panels were used and made up of no more than 19 fluorophores. Markers required for gating populations of interest were always incorporated in each panel. Fluorochrome-conjugated antibodies used for surface staining included anti-CX3CR1 (SA011F11, Biolegend), anti-I-A/I-E (M5/114.15.2, Biolegend), anti-CD45 (30-F11, Biolegend), anti-NK1.1 (PK136, Biolegend), anti-Ly6G (1A8, Biolegend), anti-CD11b (M1/70, Biolegend and BD Biosciences), anti-CD11c (HL3, BD Biosciences), anti-Ly6C (HK1.4, Biolegend), anti-CD3ε (145-2C11, Biolegend and BD Biosciences), anti-B220 (RA3-6B2, Biolegend and BD Biosciences), anti-CD115 (AFS98, Biolegend), anti-CD86 (GL-1, Biolegend), anti-CD64 (X54-5/7.1, Biolegend), anti-CCR2 (SA203G11, Biolegend), anti-F4/80 (BM8, Biolegend), anti-MerTk (2B10C42, Biolegend, DS5MMER, eBiosicence), anti-P2RY12 (S16007D, Biolegend), anti-TMEM119 (106-6, Abcam), anti-CD44 (IM7, Biolegend), anti-VLA4/CD49d (9C10, Biolegend) and anti-LFA1 (CD11a) (M17/4 or I217, Biolegend). Cells were washed twice and fixed in fixation buffer (Biolegend).

Anti-CD68 (FA-11, Biolegend), anti-CD206 (C068C2, Biolegend), anti-IFN-γ (XMG1.2, Biolegend), anti-IL-12/IL-23 p40 (C15.6, Biolegend) were stained intracellularly after surface staining, fixation and incubation with Cytofix/Cytoperm (BD Biosciences). For intracellular cytokine staining (anti-IFN-γ and anti-IL-12/IL-23 p40), cell suspensions were maintained in brefeldin A (5 μg/mL) (Sigma-Aldrich -MO, USA) until fixation.

Anti-BrdU (3D4 or Bu20a, Biolegend) was stained intranuclearly as previously described [35]. Briefly, after cell surface staining and fixation, cells were incubated in Cytofix/Cytoperm (BD Biosciences), Cytoperm Permeabilization Buffer Plus (BD Biosciences) and DNase (DN25, 30 U/sample) (Sigma-Aldrich, MO, USA), prior to being stained with anti-BrdU. For annexin V staining, cells were incubated with Annexin V (Biolegend) in Annexin V binding buffer (Biolegend) after cell surface staining. Cells were immediately analysed without fixation.

Fluorescently-tagged antibodies were measured using the FACSDiva programme on an LSR-II fluorescence-activated cell sorter (FACS) (Becton Dickinson, San Jose, CA). Expression of annexin V (Fig. 9f-h) and staining of brain cells with True-stain monocyte blocker^TM^ was measured on the 5-laser Aurora, Spectral cytometer (Cytek Biosciences). Acquired data was analysed in FlowJo (Tree Star Inc., Ashland, OR, v10.5).

Quality control gating, including time, single cells, non-debris/cells and Live/Dead staining, was applied to exclude debris, doublets and dead cells. Cell numbers were quantified using cell proportions exported from FlowJo and total live cell counts.

### Detection of nitric oxide

Detection of nitric oxide (NO) was achieved using DAF-FM diacetate (Invitrogen, USA), which is a cell-permeable compound that becomes fluorescent in the presence of NO. Prior to flow cytometric cell surface staining, cells were suspended in DAF-FM (5 nmol/mL) in PBS for 30 min at room temperature and subsequently washed. The DAF-FM was detected flow cytometrically.

### Cell sorting and adoptive transfer

Adoptive transfer and cell sorting was performed as previously described by Niewold et al. [36]. The bone marrow from dpi 5 WNV-infected mice was isolated from femurs, tibias and humeri. Two donors were used per recipient. Red blood cells were lysed using RBC lysis buffer (Biolegend). Single cell suspensions were then incubated with anti-CD16/32 and LIVE/DEAD Blue (UVLD) (Invitrogen) before being incubated with fluorescently-labelled antibodies against CD45, CD115, CD11b, Ly6C, Ly6G and B220. Ly6C^hi^ monocytes were sorted on an Influx cell sorter using the FACSDiva Programme (Becton Dickinson). The gating strategy used is shown in Niewold et al. [36]. The purity of the sorted cells was > 90% and ~3 × 10^5^ cells were transferred to each recipient. These cells were labelled with CFSE (Sigma-Aldrich, MO, USA) to the manufacturers’ instructions and injected intravenously into matched WNV dpi 5 recipients in 200 μL sterile PBS. Recipients were sacrificed on dpi 7 and brains were isolated and processed for flow cytometry, as described above.

### tSNE analysis

The FCS files were compensated and gated in FlowJo prior to exporting channel values as CSV files. CSV files were exported from either live cell, live myeloid (CD45^+^ CD11b^+^), live microglia or live non-eosinophil (SSC-A^lo^ Ly6G^−/+^) gates. t-Distributed Stochastic Neighbor Embedding (tSNE) was applied to CSV files, in RStudio (1.1.453) using the CAPX script default settings [35, 37] (package publicly available: https://github.com/sydneycytometry/CAPX). Selected markers were used to perform clustering on CD11b, CD45, F4/80, CD64, CD11c, CX3CR1, CCR2, Ly6G, Ly6C, F4/80, MHC-II, CD86, CD68, MerTK, P2RY12 and TMEM119 (in some instances not all markers were included in the FACS panel and thus were not used for clustering, thus in instances where enzyme digestion was used, we did not stain for TMEM119 and thus it was not included as a cluster parameter) using perplexity = 30, theta = 0.5 and iterations = 1000.

### Heatmaps

The FCS files were compensated and gated in FlowJo prior to exporting median fluorescent intensity (MFI) signals from populations of interest. Heatmaps were applied to MFI’s in RStudio (1.2.1335) using the Spectre script [35, 38] (package publicly available: https://github.com/ImmuneDynamics/Spectre) or with the PheatMap package [39].

### Imaging mass cytometry

Brains were isolated from mice perfused transcardially with PBS and 2% PFA. Brains were cut sagittally and placed in 2% PFA overnight, and subsequently placed sequentially in a series of sucrose solutions of increasing concentration (10%, 20%, and 30%). Murine brains were frozen in optimal cutting temperature (O.C.T.) compound (Tissue-Tek, Tokyo, Japan) in hexane pre-chilled in liquid nitrogen. Frozen brain sections (8-9 μm) were fixed in methanol, rinsed in tris-buffered saline with 0.05% Tween 20 (TBST) and blocked with 10% FCS. Excess solution was shaken off before primary fluorophore-conjugated antibodies (anti-CD11b: M1/70, Biolegend and anti-NS1: 4G4, Roy Hall, UQ) were applied. Regions of interest were selected based on immunofluorescent staining visualised on the Olympus BX-51 microscope using a DP-70 camera and Cell Sensor software. Slides were then incubated with metal-conjugated antibodies overnight at 4 °C (anti-Ly6C (HK1.4, Biolegend), anti-CD86 (GL1, Becton Dickinson), anti-Iba1 (EPR165, Abcam), anti-CD45, (30-F11, Biolegend), anti-FITC (FIT-22, DVS) and anti-NeuN (Fox3, Biolegend)). Iridium DNA intercalator (Fluidigm, 1:2000 in TBST) was applied to brain sections, prior to sections being rinsed in UltraPure water and left to air dry. If slides were not immediately ablated on the Hyperion, they were stored at 4 °C until use.

### Image generation

Once slides were imaged on the Hyperion, mass cytometry data (MCD) files were exported from the Hyperion acquisition software. For image generation, MCD files were imported into HistoCAT++ where false colour was applied to relevant channels.

### RNAse protection assay

The RNAse protection assay was performed for IL-12 (p35), IL-23 (p19), IL-12/IL-23 (p40), TNF, IL-6, IFN-γ and IL-17A. The genomic clone, RPL32-4A, a probe for the ribosomal protein L32 (provided by M. Hobbs, The Scripps Research Institute, San Diego, CA), was used as an internal control for RNA loading during RPA analysis. Briefly, total RNA was extracted from perfused brains using TRIzol reagent (Sigma-Aldrich) according to the manufacturer’s instructions. For each RPA analysis, 5 μg of RNA was used.

### IL-23 ELISA

Brains isolated from mock and WNV-infected mice were perfused, homogenised and serially diluted. The mouse IL-23 (p19/p40) ELISA MAX™ Set Deluxe (Biolegend) was used to detect IL-23 (p19/p40) production in the tissue, according to the manufacturer’s instructions.

### Statistical analysis

Non-parametric statistical tests were applied to data in GraphPad Prism 8.4.3 (GraphPad Software, La Jolla, CA). Comparison of two groups was conducted using Mann–Whitney test, and three or more groups were compared using a Kruskal–Wallis test with a Dunn’s multiple comparison test. When two independent variables and three or more groups were being compared, a two-way ANOVA and a Šídák’s or Tukey’s multiple comparisons test was used. *P* values of < 0.05 were regarded as significant and designated in figures as *P < 0.0332, **P < 0.0021, ***P < 0.0002, ****P < 0.0001. Error bars are shown as standard error of the mean (SEM).

## Results

### Classic flow cytometric gating fails to discriminate resident from infiltrating myeloid cells during severe inflammation

During viral encephalitis, the overlapping expression of cell surface markers makes the identification of resident and infiltrating myeloid cells in the CNS difficult by standard gating approaches. We therefore set out to identify the minimum parameters required to delineate these populations accurately. Cells were dissociated from murine brains at various timepoints in the progression of lethal WNE. Figure 1a-c shows a clear CD45^lo^, CD11b^hi^ microglial cell population which expresses low levels of Ly6C in the brain parenchyma of dpi 0 mice. However, from dpi 3 onwards, this discrete microglial cell population became progressively obscured by the overlapping antigen expression of increasing numbers of CD45^hi^, CD11b^hi^ and Ly6C^hi^, infiltrating BM-derived monocytes. From dpi 5-7, these monocytes showed further CD45 and CD11b upregulation with some downregulation of Ly6C (Fig. 1a-d). Compounding this, by dpi 5, microglia had also upregulated CD45 and Ly6C (Fig. 1a, b), making it impossible to separate these populations accurately by standard gating during WNE.

In order to resolve this, we used the ‘microglia-specific’ markers, TMEM119 [40] and P2RY12 [11]. However, while collagenase/DNase treatment substantially increases leukocyte yields and live:dead cell ratios during preparation of single cell suspensions (Additional file 1), TMEM119 did not label cells prepared this way (Fig. 1e, f). This has not previously been reported, to our knowledge. Ironically, without enzyme treatment, TMEM119 was downregulated on microglia at dpi 7, precluding the use of this microglia-specific marker to distinguish between resident and infiltrating myeloid cells (Fig. 1g). On the other hand, P2RY12 was unaffected by enzyme treatment, but was expressed both by a discrete CD45^int/lo^ population at day 0 and on different populations of CD45^int/hi^ cells at dpi 7 (Fig. 1h). Thus, it was also unclear whether this purinergic receptor, putatively expressed only by microglia, was also expressed by an infiltrating CD45^hi^ macrophage population. It should be noted that collagenase/DNase was used in the remainder of this study for optimal brain cell preparation, except where TMEM119 was measured.

### High parameter cytometry and dimensionality-reduction can delineate resident and infiltrating myeloid cells

As shown above, the discrimination of microglia from infiltrating myeloid cells in severe neuroinflammatory conditions is unreliable, even with the use of ‘microglia-specific’ markers. Other groups have distinguished microglia from macrophages based on the higher macrophage expression of CD44 [41, 42], VLA4 (CD49d) [43], LFA1 (CD11a) [44], CCR2 and Ly6C. In WNE, the expression profile of these markers on some infiltrating myeloid cells, viz. Ly6C^hi^CD45^int^ and Ly6C^int^CD45^int^, was similar to microglia in the infected brain (Additional file 2). Thus, we were also unable to distinguish these populations using these markers. To address this issue, we labelled cells for myeloid cell markers (CD45, CD11b, F4/80), ‘activation/functional’ myeloid markers (CD11c, MerTK, CD64, CD68 CD86, MHC-II), ‘infiltrating/inflammatory macrophage’ markers (CCR2, Ly6C) and markers ‘specific’ for and/or highly expressed on microglia (P2RY12, TMEM119, CX3CR1) and analysed them by fluorescence flow cytometry. We visualised the acquired high parameter data on a 2D plot after subjecting it to t-Distributed Stochastic Neighbor Embedding (t-SNE), which clusters cell populations based on similarity of marker expression [45]. We then generated a series of gating strategies to identify discrete populations clustered on the tSNE plot.

**Fig. 2:**
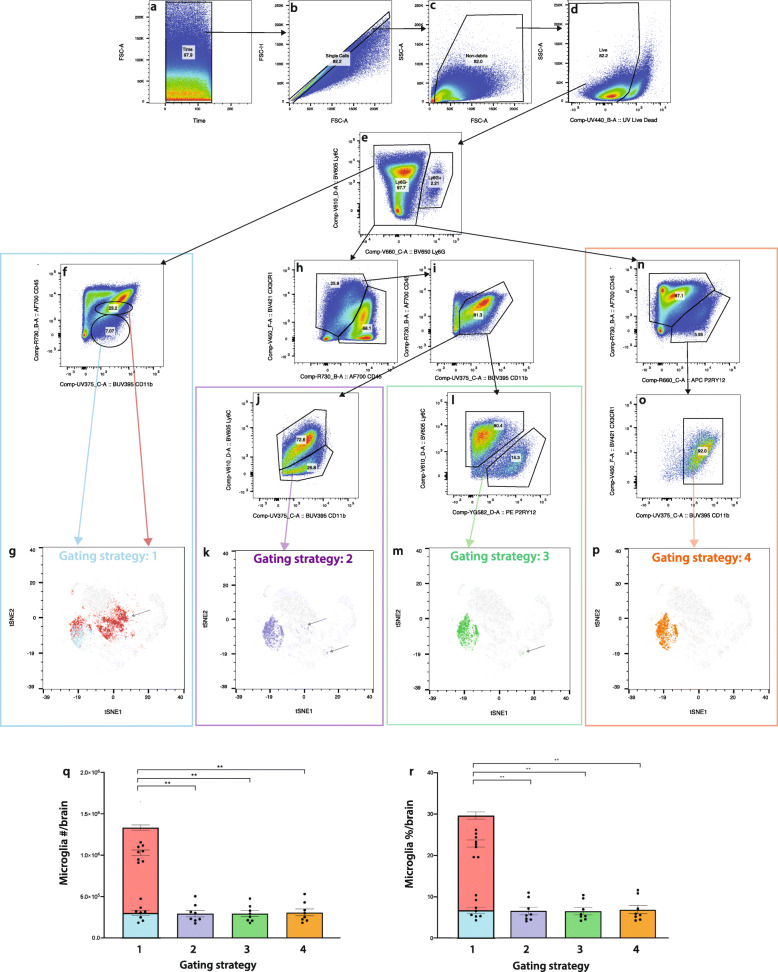
A revised gating strategy can be used to identify microglia in WNV-infection. **a-d** Quality control gates, including time (**a**), single cells (**b**), non-debris (**c**) and live cell (**d**) gates were applied before analysing cells. Neutrophils were also excluded by their expression of Ly6G (**e**). **f** Gating strategy one identifies a ‘resting’ microglia population (CD45^lo^ CD11b^+^) (R1: blue) and an ‘activated’ microglia population (CD45^int^ CD11b^+^) (R2: red). **h**, **j** Gating strategy 2, does not use ‘microglia-specific markers’ and identifies microglia as CX3CR1^+^ CD45^lo-int^ CD11b^+^ Ly6C^−/lo^. Gating strategy 3 is a revised gating strategy which identifies microglia as CX3CR1^+^ CD45^lo-int^ CD11b^+^ P2RY12^+^ Ly6C^−/lo^ (**h**, **i**, **l**). **n**, **o** Gating strategy 4 uses a limited number of markers and identifies microglia as CD45^lo-int^ P2RY12^+^ CD11b^+^ CX3CR1^+^. **g**, **k**, **m**, **p** ‘Microglia’ populations gated using strategies 1 (**g**), 2 (**k**), 3 (**m**) and 4 (**p**), overlaid onto a tSNE plot, clustered on live cells from WNV dpi 7 brains. **q**, **r** Number (**q**) and frequency (**r**) of ‘microglia’ gated using strategies 1-4. Data is presented as mean ± SEM, from two independent experiments with at least six mice per group. **P < 0.0021, Kruskal-Wallis test and Dunn’s multiple comparisons test

In the homeostatic brain, four different fluorescence gating approaches effectively identified the same microglial population (Additional file 3). However, in severe neuroinflammation (Fig. 2), these approaches produced different results. Gating strategy 1 identified ‘resting’ microglia as CD45^lo^ CD11b^+^ and ‘activated’ microglia as CD45^int^ CD11b^+^ (Fig. 2f). This strategy has typically been used to identify microglia in both the homeostatic and diseased brain [1, 46, 47]. The CD45^lo^ CD11b^+^ ‘resting’ microglia gate comprises a single cluster when overlaid onto a tSNE plot from WNV dpi 7 brains (Fig. 2g, blue). However, the CD45^int^ CD11b^+^ gate captures infiltrating monocytes/macrophages in neuroinflammatory and neurodegenerative models [1, 48]. Accordingly, the ‘activated’ microglia gate comprises 2 distinct clusters on the tSNE plot (Fig. 2g, red).

We therefore generated and compared further gating strategies to determine the feasibility of more accurately distinguishing resident from infiltrating myeloid populations (Fig. 2). Microglia were identified as Ly6G^−^, CX3CR1^+^, CD45^lo/int^, CD11b^+^, Ly6C^−/lo^ (gating strategy 2, Fig. 2j, k); Ly6G^–^, CX3CR1^+^, CD45^lo/int^, CD11b^+^, P2RY12^+^, Ly6C^−/lo^ (gating strategy 3, Fig. 2l, m) and Ly6G^−^, CD45^int/lo^, PR2Y12^+^, CX3CR1^hi-lo^, CD11b^+^ (gating strategy 4, Fig. 2o, p). Strategies 2 and 3 resulted in the inclusion of various populations outside of the putative microglial cluster in the tSNE plot, while strategy 4 identified a single microglial cluster with very little contamination from other cells. Strategy 4 used the fewest markers and gates, the simplest gating, and was thus least susceptible to error. We employed gating strategy 4 to further investigate the identities and change kinetics of these populations during WNE. Nevertheless, since strategies 2 and 4 gave putative microglial numbers that were not statistically different (Fig. 2q, r), previous data using standard markers prior to the availability of P2RY12 could be re-analysed more accurately using strategy 2.

### Modulating CNS infiltration verifies non-microglial populations in gating strategy 4

In order to confirm that gating strategy 4 was accurately distinguishing resident from infiltrating myeloid populations during WNE, we employed 3 approaches. We (1) blocked the entry of myeloid populations infiltrating into the brain from peripheral blood, (2) adoptively transferred BM-derived monocytes and tracked their phenotypic changes after infiltration and (3) injected intravenous dye to label peripheral leukocytes that infiltrate the brain.

**Fig. 3:**
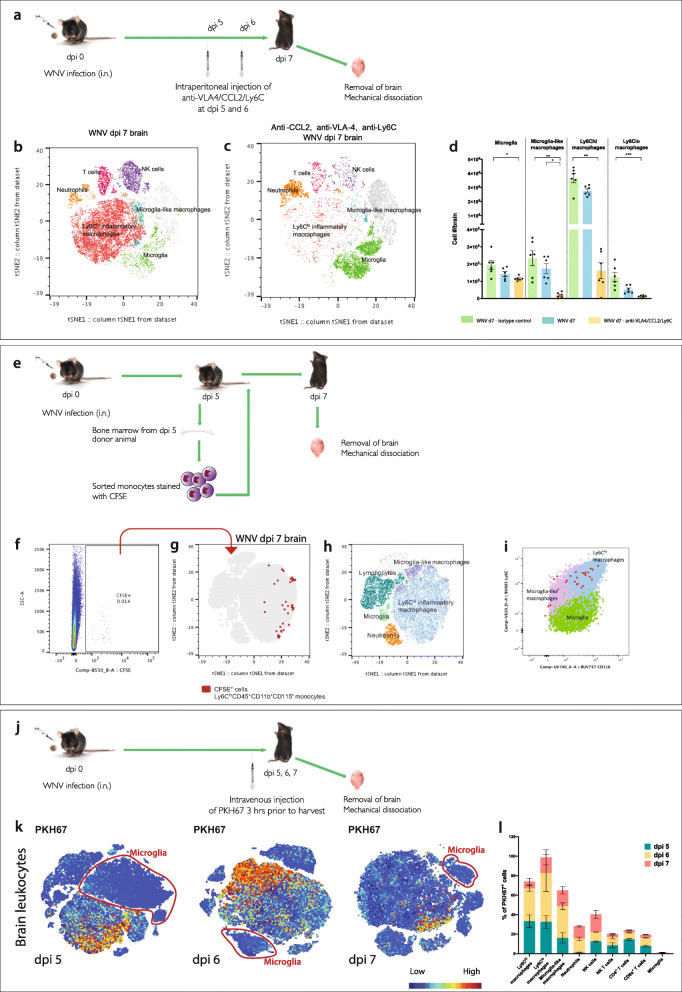
Adoptively transferring, staining and blocking infiltrating monocytes support the use of our revised gating strategy. **a** Schematic showing the injection regimen used to block entry of monocytes in WNV-infected brains. Mice were treated intraperitoneally (i.p.) with a cocktail of blocking antibodies (anti-VLA4/CCL2/Ly6C) at dpi 5 and 6. **b**, **c** tSNE plot representing WNV dpi 7 brains from mice without blockade treatment (**b**) and with blockade treatment (**c**). **d** Number of microglia, Ly6C^hi^, Ly6C^lo^ and microglia-like macrophages in WNV dpi 7 brains from non-treated animals and animals treated with isotype control antibodies or anti-VLA4/Ly6C/CCL2 blocking mAbs. Data is presented as mean ± SEM, with six mice per group, from two independent experiments. *P < 0.0332, **P < 0.0021, ***P < 0.0002, Kruskal-Wallis test and Dunn’s multiple comparisons test. **e** Schematic of adoptive transfer workflow. Ly6C^hi^ CD45^+^ CD11b^+^ CD115^+^ BM monocytes were sorted from WNV dpi 5 animals, stained with CFSE and transferred intravenously into matched WNV dpi 5 recipients. Brains were collected at dpi 7, and cells were isolated for FACs analysis. **f** FACs plot of live, CFSE^+^ cells from a recipient animal at WNV dpi 7. **g** CFSE^+^ cells overlaid onto a tSNE plot clustered on live cells from recipient animals at WNV-dpi 7. tSNE plot annotated with myeloid and lymphoid populations (**h**), showing that CSFE^+^, do not fall into the putative microglia population. **i** FACs plot showing the expression of CD45 and CD11b on CFSE^+^ transferred cells, microglia-like macrophages, Ly6C^hi^ macrophages and microglia. **j** Diagram showing the injection regimen used to stain cells infiltrating WNV-infected brains. Mice were injected intravenously with PKH67 at dpi 5, 6 and 7, 3 h prior to harvest. **k** tSNE plots representing WNV dpi 5, 6 and 7 brains from mice injected with PKH67, each generated separately. Microglial cells are circled in red and do not exhibit PKH67 staining

Infiltration of cells into the brain parenchyma was blocked using an antibody cocktail made up of anti-VLA4 [3], CCL2 [1]  and Ly6C [49] at dpi 5 and 6 (Fig. 3a). This resulted in a 90-95% reduction in the number of infiltrating Ly6C^hi^ inflammatory and a population of ‘microglia-like’ macrophages (i.e.*,* non-microglial myeloid cells present in the infected brain with a CD45^+^, CX3CR1^+^ and CD11b^+^ profile similar to microglia, but absent in the homeostatic brain) (Fig. 3b-d). This was associated with a commensurate depletion in the corresponding areas of the tSNE plot (Fig. 3b). Non-microglial populations shown in the tSNE plot (Fig. 3b, c) were identified using the gating strategy shown in Additional file 4. A heatmap showing the immune profiles of the identified myeloid populations are further shown in Additional file 5. Importantly, the antibody cocktail did not reduce numbers of putative microglia in animals treated with blocking antibodies, compared to untreated animals (Fig. 3b-d). This suggests that gating strategy 4 identifies microglia, even during highly inflammatory conditions.

Unexpectedly, the use of the isotype control antibody cocktail increased the number of microglia and infiltrating macrophages, compared to untreated mice (Fig. 3d), raising the question of whether some of the infiltrating cells were falling into the putative microglia gate. To investigate this, we adoptively transferred CFSE^+^ BM-derived CD115^+^, CD45^+^, CD11b^+^, Ly6C^hi^, Ly6G^−^ and B220^−^ monocytes from dpi 5 WNV-infected mice, into recipient age-, sex- and time-matched WNV-infected animals and harvested the recipient brains on dpi 7 (Fig. 3e). Analysis shows that all of the transferred cells fell outside the identified putative resident microglia tSNE cluster (Fig. 3f-h) and appeared in both the infiltrating Ly6C^hi^ inflammatory and ‘microglia-like’ macrophage gates (Fig. 3i).

Considering only a small number of transferred cells could be tracked via adoptive transfer, we also injected mice with PKH67 3 h prior to collecting brain tissue, to definitively distinguish infiltrating from resident myeloid populations (Fig. 3j, k). At dpi 5, 6 and 7, the majority of PKH67^+^ cells infiltrating WNV-infected brains were infiltrating monocytes or lymphocytes, while cells in the putative microglial cluster showed no staining. Taken together, the data strongly suggests that this cluster only represents the resident microglia.

### Microglia adopt ‘disease-specific’ immunophenotypes in WNE

**Fig. 4:**
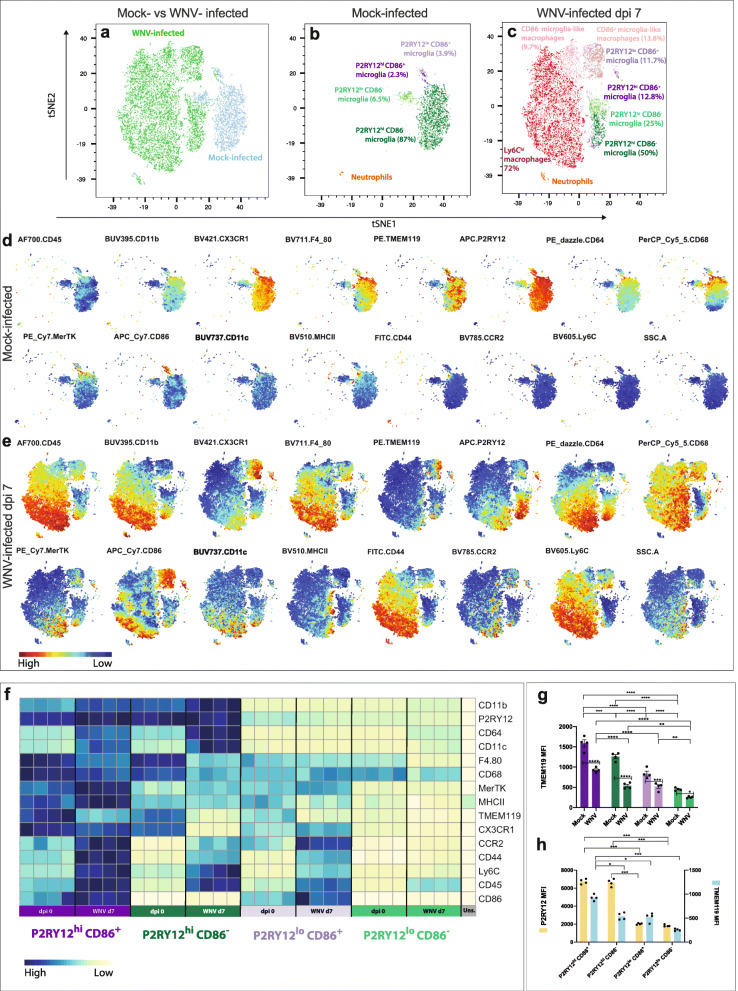
Dimensionality reduction analysis reveals four microglia subsets that adopt distinct phenotypes in WNE. **a-c** tSNE plots clustered on live myeloid cells (UVLD^−^ CD11b^+^ CD45^+^) from WNV dpi 0 and 7 murine brains. Mock-infected and WNV dpi 7 cells overlaid onto tSNE plots (**a**). Annotation of myeloid populations gated and overlaid onto tSNE plots representing mock-infected (**b**) and infected (**c**) brains. tSNE plots showing the relative intensity of selected markers/parameters in mock-infected (**d**) and infected brains (**e**). **f** Heatmap showing the expression of selected markers in P2RY12^hi^ CD86^+^, P2RY12^lo^ CD86^−^, P2RY12^lo^ CD86^+^ and P2RY12^hi^ CD86^−^ microglia and unstained (uns.) brain cells. **g** MFI of TMEM119 on 4 microglia populations in mock-infected and WNV-infected brains. **h** Comparison of P2RY12 and TMEM119 MFI on four microglia populations in WNV-infected brains. Data is representative of at least two independent experiments and presented as mean ± SEM, with four mice per group. *P < 0.0332, **P < 0.0021, ***P < 0.0002, ****P < 0.0001, two-way ANOVA with a Šídák’s multiple comparisons test

Using gating strategy 4, we then proceeded to investigate the immunophenotypic heterogeneity and response of microglia during WNE. We pre-gated live, CD11b^+^, CD45^+^ myeloid cell populations and ran tSNE analysis on the concatenated dpi 7 WNV-infected and mock-infected populations (Fig. 4a-c).

Clustering the pre-gated cells on CD45, CD11b, CX3CR1, CD64, F4/80, CD86, CD68, P2RY12, Ly6C, CD44, MHC-II, CD11c, CCR2, MerTK and TMEM119, revealed 4 distinct microglial phenotypes in both the homeostatic and infected brain (Fig. 4b, c). These were, PR2Y12^h^^i^ CD86^−^, PR2Y12^hi^ CD86^+^, PR2Y12^lo^ CD86^−^ and PR2Y12^lo^ CD86^+^. Importantly, gating strategy 4 could identify these subsets in enzyme- and non-enzyme-digested brains, as this approach does not rely on TMEM119 or CD44 detection, which is reduced on digestion (Additional file 6).

Strikingly, the microglial cluster in the homeostatic brain was in a different position on the tSNE plot from that in the infected brain. This indicates a substantial phenotypic change in microglia during infection (Fig. 4a-c). Consistent with this, at dpi 7, all microglia phenotypes had downregulated TMEM119, CX3CR1, F4/80 and CD68 and upregulated CD45 and CD64 (Fig. 4d-f and Additional file 7). However, in contrast to the significant downregulation of TMEM119, P2RY12 expression was relatively stable from dpi 0 to 7 (Fig. 4d-h). Notwithstanding these phenotypic changes, the same four distinct subsets could be identified throughout the disease course.

The P2RY12^hi^ microglia subsets in both mock-infected and infected brain had higher expression of all measured markers, compared to the P2RY12^lo^ subsets (Fig. 4f-h). P2RY12^hi^ cells from infected brains upregulated CD45, CD11b, CD64, MerTK, CD11c, CD44, CCR2 and Ly6C, relative to microglia at dpi 0. Of the P2RY12^hi^ population, the CD86^+^ microglia showed the highest expression of CX3CR1, F4/80, TMEM119, CD68, MerTK, MHC-II, CD44, CCR2 and Ly6C, while CD86^−^ microglia showed the highest expression of CD11b, CD64 and CD11c. Notably, microglia that expressed the highest levels of ‘activation’ markers in the infected brain paradoxically also had the highest expression of nominally homeostatic markers, CX3CR1, TMEM119 and P2RY12.

In contrast to microglia, infiltrating myeloid cells in these tSNE plots fell into 2 principal populations, Ly6C^hi^ inflammatory macrophages (CD45^hi^, CD11b^hi^, CX3CR1^lo^, F4/80^+^, CD64^hi^, CD68^hi^, CD44^hi^, Ly6C^hi^, 72.5%) and microglia-like macrophages (CD45^int^, CD11b^int^, CX3CR1^hi/int^, F4/80^+^, CD64^int^, CD68^hi^, CD44^int^, Ly6C^int^, 23.5%) (Fig. 4e). Both populations had varied expression of MerTK, CD86, CD11c, MHC-II and CCR2, as well as TMEM119 and P2RY12. Since peripheral myeloid populations in the blood and bone marrow expressed neither TMEM119 nor P2RY12 (Additional files 8 and 9), this indicates that myeloid cells upregulated these ‘microglia-specific’ markers, de novo after CNS infiltration.

### Microglial phenotypes depleted by PLX5622 are not brain region-specific

**Fig. 5:**
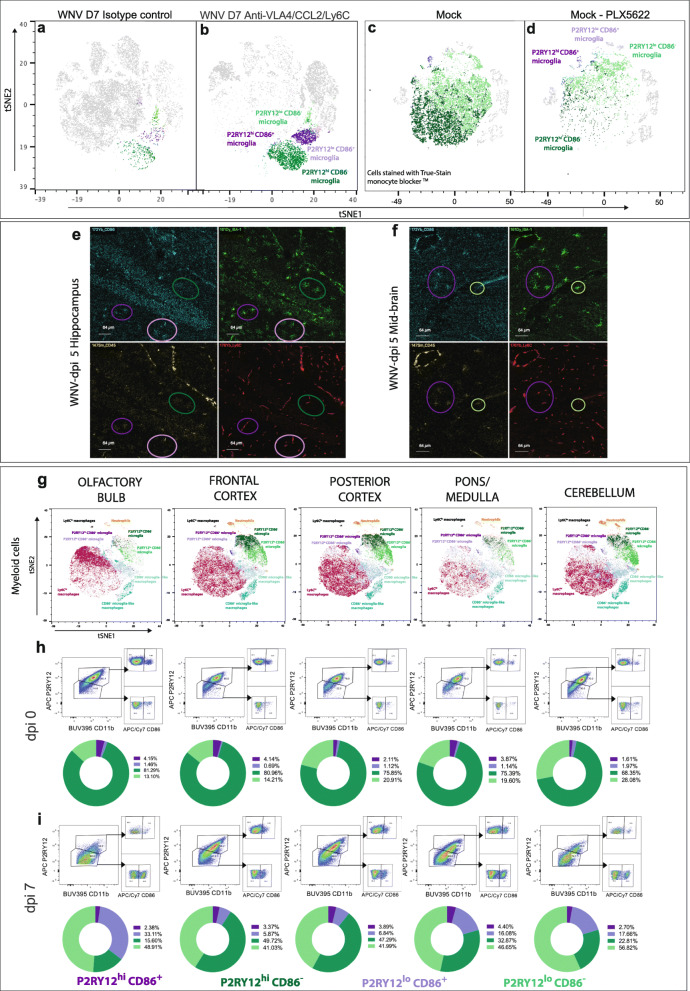
The identified microglial subsets are not peripherally derived nor represent region-specific phenotypes. **a-d** Microglia ‘subsets’ gated and overlaid onto tSNE plots representing WNV dpi 7 brains from animals treated with isotype (**a**) and VLA4/CCL2/Ly6C blockade antibodies (**b**) and mock-infected brains from animals treated with (**d**) and without (**c**) PLX5622. **e**, **f** IMC images of the hippocampus (**e**) and mid-brain (**f**) from a WNV dpi 5 animal, showing the expression of CD45 (cyan), Iba-1 (green), CD86 (yellow) and Ly6C (red). Circles show the different microglia ‘subsets’ P2RY12^hi^ CD86^+^ (dark purple), P2RY12^lo^ CD86^−^ (light green), P2RY12^lo^ CD86^+^ (light purple), P2RY12^hi^ CD86^−^ (dark green). **g** tSNE plots clustered on live myeloid cells (UVLD^−^ CD11b^+^ CD45^+^) from WNV dpi 7 brains, segmented into 5 anatomical regions. Myeloid populations from the olfactory bulb, frontal cortex, posterior cortex, pons/medulla and cerebellum were gated and overlaid onto the plot. **h**, **i** FACs plots and pie graphs showing the relative proportions of each microglia ‘subset’ found in 5 anatomical regions of non-infected (**h**) and WNV-infected brains (**i**). Values on pie graphs indicate the frequency of each microglial population out of total microglia. Data shown in **a-d** is representative of two independent experiments with six mice in each group. Data shown in **g-i** is representative of one experiment with six brains pooled per brain region

Abrogating myeloid and lymphoid cell infiltration, using systemic antibody blockade, did not reduce microglial numbers (Figs. 3d, 5a, b). In contrast, the 4 phenotypes defined by CD86 and P2RY12 expression were substantially and proportionally decreased in animals treated with chow containing CSF1R inhibitor, PLX5622. (Fig. 5c, d). Taken together, these data strongly suggest that these cells are resident microglia.

There have been limited reports of a CD86-expressing microglial population in the homoeostatic or infected brain. Therefore, we first established that the expression of CD86 was not a result of the reported non-specific binding of cyanine dyes (APC-Cy7 – Fig. 4) to macrophages [50] (Additional file 10). To further confirm this, we examined brain sections for a CD86^+^ microglia using metal-labelled antibodies in imaging mass cytometry (IMC). At dpi 5 with minimal myeloid cell infiltration, microglia were readily identifiable by their ramified morphology and Iba1^+^ and Ly6C^−^ expression profile. These cells were CD86^+^ in various regions of the brain (Fig. 5e, f).

**Fig. 6:**
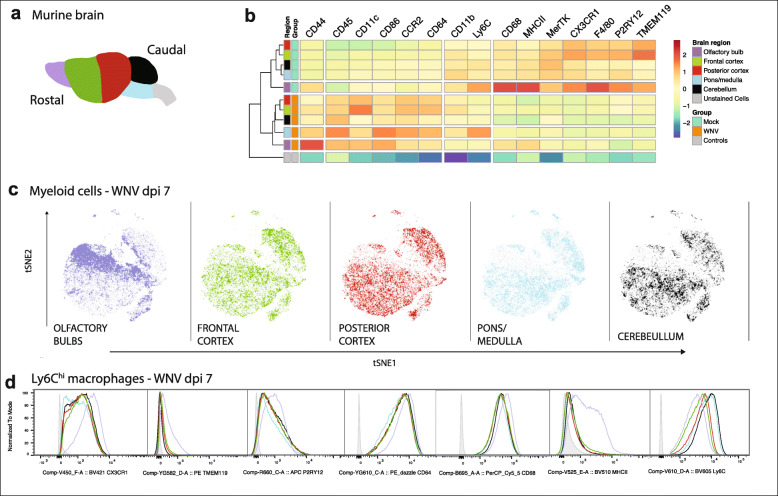
Resident and infiltrating myeloid cells show spatial immunophenotypic heterogeneity in WNE. **a** Schematic showing the 5 anatomic regions dissected from the murine brain. **b** Heatmap showing the relative intensity of selected markers on/in unstained (uns.) cells and microglia in the olfactory bulb, frontal cortex, posterior cortex, pons/medulla and cerebellum in the homeostatic and WNV-infected brain. **c** tSNE plot clustered on live myeloid cells from all brain regions and overlaid with total myeloid cells found in each region: olfactory bulb, frontal cortex, posterior cortex, pons/medulla and cerebellum. **d** Histograms showing signal intensities of selected markers on Ly6C^hi^ macrophages from 5 different brain regions in WNV dpi 7 brains. Unstained cells are represented by the shaded grey peak in each plot. Data shown in **a-d** is representative of one experiment with six brains pooled per brain region

In dissecting the brain into 5 separate regions, viz. olfactory bulb, frontal cortex, posterior cortex, pons/medulla and cerebellum, the 4 identified microglial phenotypes were found in all areas by flow cytometry (Fig. 5g). This indicates that these phenotypes are not region-specific and supports the IMC data. Nevertheless, there was significant variation in the proportion of each microglial subset between these anatomical areas, both under homeostatic conditions and in response to WNV infection (Figs. 5h, i, 6b). Under homeostatic conditions, the largest group was the CD86^−^ microglia, with the P2RY12^hi^ subset comprising 68-80% and the P2RY12^lo^ subset, 13-28%. The CD86^+^ subsets together comprised less than 5% of microglia. In response to WNV, there was an increase in the proportions of both P2RY12^lo^ subsets in all anatomical areas by dpi 7, principally at the expense of the P2RY12^hi^CD86^−^ (Fig. 5h, i).

Microglia in the olfactory bulbs had the highest expression of CX3CR1, F4/80, CD68 and MHC-II in both the homeostatic and infected brain. However, in WNV-infected brains they had the lowest expression of CD11b, TMEM119 and P2RY12 of all the anatomical sites (Fig. 6a-b). Considering WNV enters the olfactory bulb and remains there over the course of infection, downregulation of these markers in this model could be a result of prolonged neuronal infection and exposure to neuroinflammation. Downregulation of TMEM119 and P2RY12 has been reported in a number of chronic neuroinflammatory models [51–53].

Considering the demonstrable progression of infection from rostral to caudal over time, it was of interest to determine changes in marker expression on microglia in the cerebellum. The cerebellum shows limited neuronal infection at dpi 7, despite a broad interferon-stimulated gene response [54]. Cerebellar microglia had a higher CD11b and MerTK expression and lower CX3CR1, F4/80, CD68, and MHC-II expression than the olfactory bulb. Nevertheless, proportions of both P2RY12^lo^ subsets were similarly increased in response to WNE in both regions (Fig. 5h, i).

More striking was the differential profile of Ly6C^hi^ macrophages in these brain regions at WNV dpi 7 (Fig. 6c, d). In regions with more prolonged viral infection (i.e.*,* the rostral region of the brain), infiltrating Ly6C^hi^ macrophages had lower Ly6C expression and higher expression of CX3CR1, TMEM119, P2RY12, CD64, CD68 and MHC-II, compared to those in the caudal regions. Presumably, these cells were recruited earlier in infection and modulated these markers, assuming a phenotype similar to microglia with increasing time spent in the brain. Nonetheless, resident and infiltrating cells in all regions remained distinct, clustering in separate groups on the tSNE plot (Figs. 5g, 6c).

### Temporal changes in microglial phenotypes during WNE

**Fig. 7:**
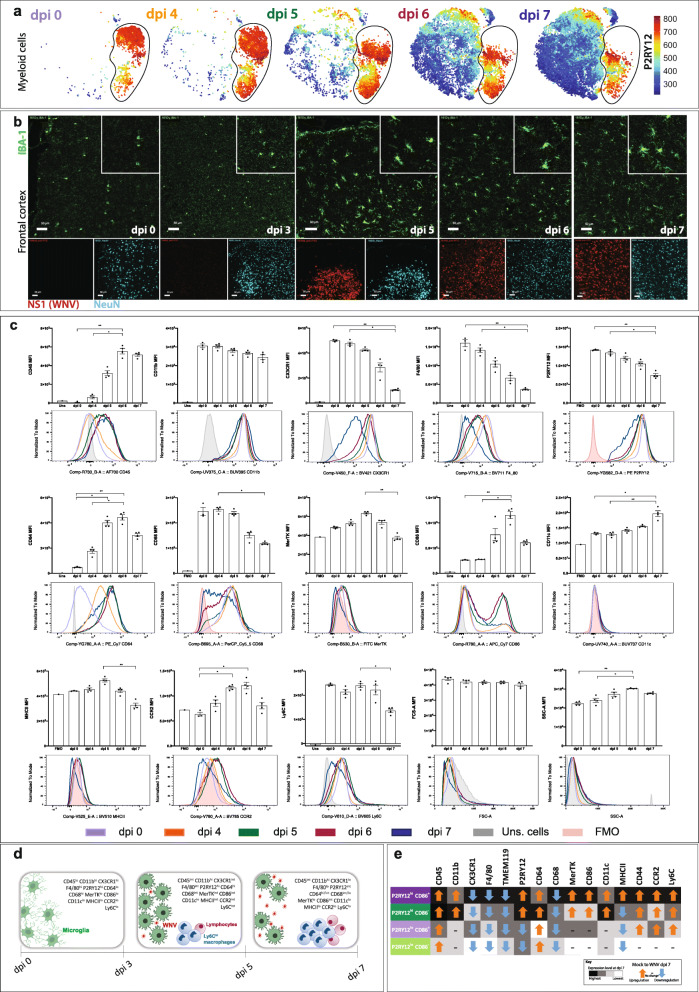
Temporal changes in microglial phenotypes in WNE. **a** tSNE plots showing the relative expression of P2RY12 on live myeloid cells from WNV-infected brains at dpi 0, 4, 5, 6 and 7. **b** IMC images from the frontal cortex of WNV dpi 0, 3, 5, 6 and 7 animals, showing the expression of Iba-1 (myeloid cells) (green), NS1 (WNV) (red) and NeuN (neurons) (blue). Scale bar represents 50 mm. **c** Histograms and statistical analysis of median fluorescence intensity (MFI) changes of selected markers/parameters on/in microglia at dpi 0, 4, 5, 6 and 7. Grey peaks/bars show unstained (uns.) cells, while pink peaks/bars show fluorescence minus one (FMO) for the respective marker. Data is representative of at least three independent experiments and presented as mean ± SEM, with 3-4 mice per group. *P < 0.0332, **P < 0.0021, Kruskal-Wallis test and Dunn’s multiple comparisons test. **d** Schematic showing microglia morphological and immunophenotypical changes upon WNV and monocyte infiltration during WNE. **e** Schematic showing the overall up- (orange arrow) or downregulation (blue arrow) of selected markers on P2RY12^hi^ CD86^+^ (dark purple), P2RY12^lo^ CD86^−^ (light green), P2RY12^lo^ CD86^+^ (light purple), P2RY12^hi^ CD86^−^ (dark green) microglial subsets from dpi 0 to dpi 7. Comparative levels of marker expression (black graded to white) between microglial subsets are shown for dpi 7. Microglial populations with a highest final expression of each marker (relative to the other 3 microglia subsets) are black. Note, this does not take account of any peak changes at dpi 5 and 6

The microglial phenotypes we identified in the naïve and WNV dpi 7 brain were also present at dpi 4-6 of WNE. With increasing infection in the brain, all microglia showed significant temporal phenotypic changes (Fig. 7a-e). Measured markers were progressively (a) upregulated, (b) downregulated or (c) upregulated and then downregulated, indicating changes in potential activity and functions at different disease stages (Fig. 7a, c-e).

From dpi 4, the total microglia population upregulated CD45, increased their granularity (side scatter - SSC-A) and progressively downregulated CX3CR1, F4/80 and CD68 over the course of infection. At dpi 5 and 6, microglia showed peak expression of several cell surface markers, including CD64, MerTK, CD86, MHC-II, CCR2 (low levels) and Ly6C, which were subsequently downregulated by dpi 7 (Fig. 7c-e). While P2RY12 was expressed by all microglial subsets, average expression was reduced on the total microglial population by dpi 7. This was due to a combination of P2RY12 downregulation only on P2RY12^lo^ cells and an increase in the proportion of this subset over the course of infection.

In the 4 individual microglial cell phenotypes identified during infection (Figs. 4f, 7e and Additional file 11), the P2RY12^hi^ cells showed a higher expression of all measured markers, compared to P2RY12^lo^ cells. While all phenotypes upregulated CD45 and CD64, the P2RY12^hi^ cells only upregulated CD11b, P2RY12 and CD11c. In contrast, P2RY12^lo^ cells downregulated P2RY12, as mentioned above, and showed no change in CD11b or CD11c expression. Of note, the P2RY12^hi^CD86^−^ cells upregulated CD86 during infection, becoming P2RY12^hi^CD86^lo^ microglia (Figs. 5i, S5 and S9).

Changes in microglial immunophenotypes correlated with monocyte infiltration and neuronal infection from dpi 4. Microglia also exhibited a reactive morphology by dpi 5, with hypertrophied cell somata and shortened cytoplasmic extensions (Fig. 7a, b). This was similar in other brain regions, irrespective of the presence of virus (data not shown).

### Microglia proliferate early and die late in infection

**Fig. 8:**
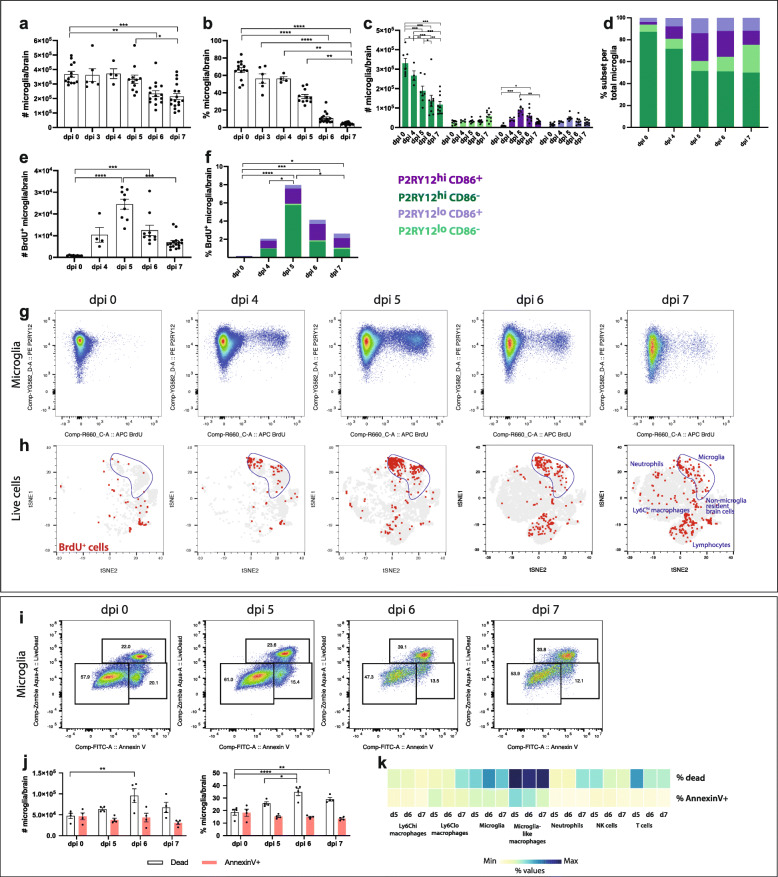
Kinetics of microglia proliferation and death in the progression of lethal encephalitis. **a**, **b** Number (**a**) and percent (**b**) of total microglia in murine brains at dpi 0, 3, 4, 5, 6 and 7. *P < 0.0332, **P < 0.0021, ***P < 0.0002, ****P < 0.0001, Kruskal-Wallis test and Dunn’s multiple comparisons test. **c**, **d** Number (**c**) and proportion (**d**) of P2RY12^hi^ CD86^+^ (dark purple), P2RY12^lo^ CD86^−^ (light green), P2RY12^lo^ CD86^+^ (light purple) and P2RY12^hi^ CD86^−^ (dark green) microglia at WNV dpi 0, 4, 5, 6 and 7. *P < 0.0332, **P < 0.0021, ***P < 0.0002, ****P < 0.0001, two-way ANOVA with a Tukey’s multiple comparisons test. Data is presented as mean ± SEM and representative of 2-3 independent experiments. **e**, **f** Number (**e**) and percent (**f**) of P2RY12^hi^ CD86^+^ (dark purple), P2RY12^lo^ CD86^−^ (light green), P2RY12^lo^ CD86^+^ (light purple) and P2RY12^hi^ CD86^−^ (dark green) microglia that are BrdU^+^ in WNV-infected brains. *P < 0.0332, **P < 0.0021, ***P < 0.0002, two-way ANOVA with a Tukey’s multiple comparisons test. **g** FACs plots showing the expression of P2RY12 and BrdU on/in microglia in murine brains in WNE. **h** tSNE plots clustered on live cells from WNV-infected brains and overlaid with BrdU^+^ (proliferating) cells (red), which were determined with a fluorescence minus one (FMO) sample (i.e.*,* no anti-BrdU staining). Microglia are outlined in blue on each plot. **i** FACs plots showing microglial cell expression of UVLD and Annexin V in WNV-infected brains. Values on FACs plots indicate the proportion of UVLD^−^ Annexin V^−^, UVLD^−^ Annexin V^+^ and UVLD^+^ Annexin V^+^ microglia out of the total microglia population. **j** Number and percent of Annexin V^+^ and dead microglia in WNV-infected brains. **k** Heatmap showing the percent of UVLD^+^ and Annexin V^+^ myeloid and lymphoid populations in murine brains over the course of WNV. Data is presented as mean ± SEM and representative of at 1-2 independent experiments, with at least 4 mice per group

Using gating strategy 4, kinetic analysis revealed a decrease in the number of total microglia later in infection, with their proportions reducing dramatically due to the increasing numbers of other leukocytes immigrating into the brain (Fig. 8a, b). Within this population, the number and proportion of the P2RY12^hi^CD86^−^ microglial subset decreased against an increase in the number and proportion of the other subsets from dpi 4-5 (Fig. 8c, d). Furthermore, notwithstanding the lack of significant change in microglial cell numbers early in infection, microglia proliferated from dpi 4, as shown by the incorporation of BrdU (Fig. 8e-i). At dpi 5, the peak of microglial cell proliferation, P2RY12^hi^CD86^−^ microglia showed the greatest incorporation of BrdU (Fig. 8g). From dpi 5-7, microglial proliferation decreased (Fig. 8e-i), whilste the frequency of lymphocyte proliferation increased over this time, and MDM proliferation was minimal (Fig. 8h). Increased myeloid cell numbers, in WNV-infected brains, was previously attributed to the infiltration of BM-derived monocytes [1, 33]; however, our analysis shows both clear microglial cell proliferation and MDM infiltration.

Coinciding with reduced microglial cell numbers by dpi 7, there was also an increased number and percentage of dead microglia (Annexin V^+^, Live/Dead stain^+^) from dpi 6 onwards. However, the proportions of apoptotic microglia (Annexin V^+^ only) were not significantly different over this time (Fig. 8j-k). Strikingly, the population with the highest proportion of dead and dying cells at dpi 5, 6 and 7 were microglia-like macrophages (Fig. 8k). The increased number of dead microglia explains, at least in part, why proliferation of microglia at dpi 5 did not correspond to an increase in microglial numbers at dpi 6 or 7.

### Microglia are the principal producers of IL-12 during lethal WNE

**Fig. 9:**
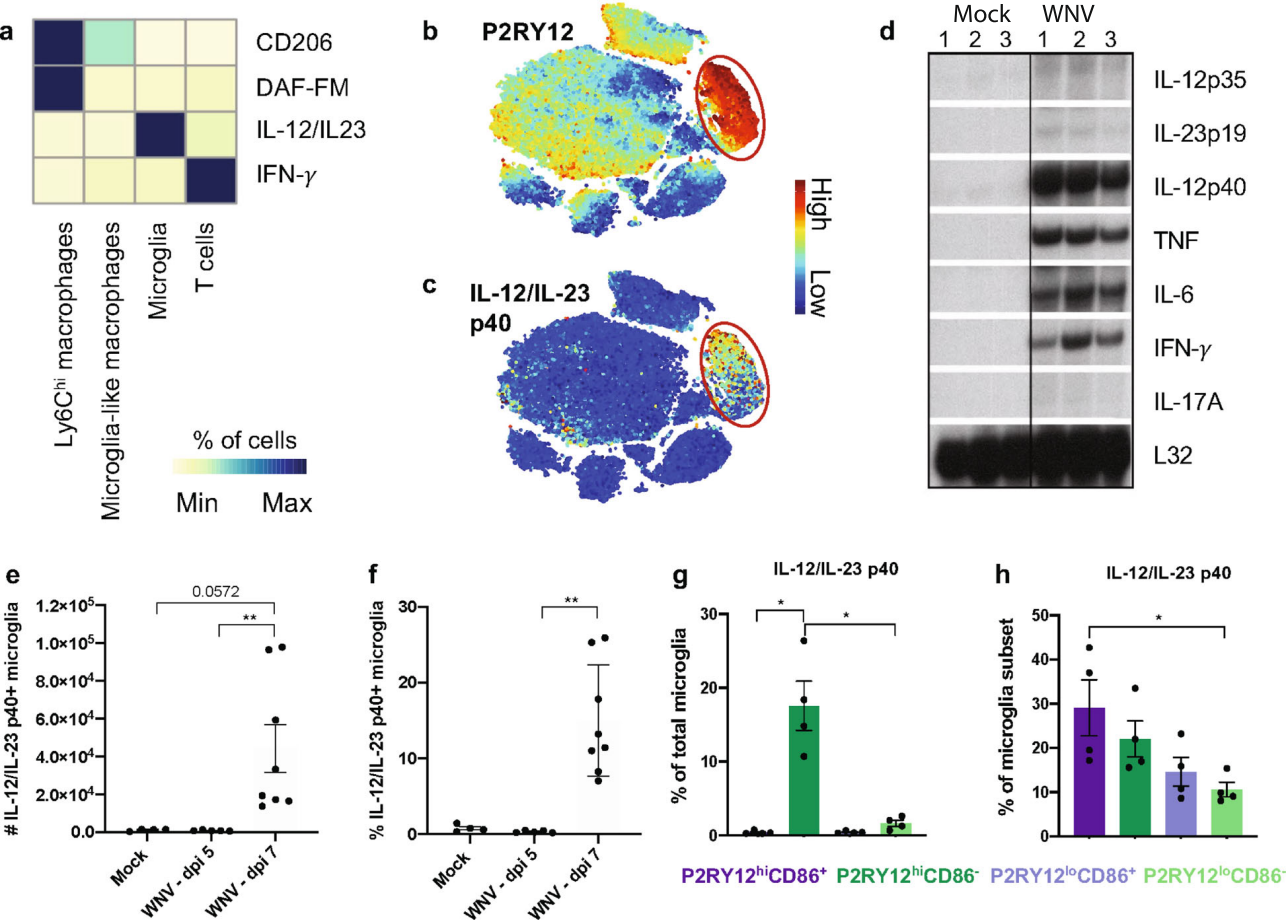
Microglia are the principal producers of IL-12 late in WNV-infection. **a** Heatmap showing the proportion of cells that are IFN-γ^+^, IL-12/IL23 (p40)^+^, DAF-FM^+^ or CD206^+^ in WNV-infected dpi 7 brains. **b**, **c** tSNE plots showing the expression of P2RY12 (**b**) and IL-12/IL-23 (p40) (**c**) on live cells isolated from WNV-infected brains at dpi 7. Red circles indicate where microglia are positioned on plots. **d** RNAse protection assays (RPA) performed for IL-12 (p35), IL-23 (p19), IL-12/IL-23 (p40), TNF, IL-6, IFN-γ and IL-17A on mock and WNV-infected brains at dpi 7. Lanes 1-3 indicate individual animals in each group. RPL32-4A was used as an internal control for RNA loading during RPA analysis. **e**, **f** The number (**e**) and proportion (**f**) of total microglia expressing IL-12/IL-23 (p40) at dpi 0, 5 and 7. **g**, **h** Proportion of P2RY12^hi^ CD86^+^ (dark purple), P2RY12^lo^ CD86^−^ (light green), P2RY12^lo^ CD86^+^ (light purple) and P2RY12^hi^ CD86^−^ (dark green) expressing IL-12/IL-23 (p40) at WNV dpi 7 out of the total microglia population (**g**) and within microglia sub-populations (**h**). *P < 0.0332, **P < 0.0021, Kruskal-Wallis test and Dunn’s multiple comparisons test

To elucidate the function and contribution of microglia to protective or pathogenic responses in WNE, we stained for a series of intracellular cytokines readily detected without in vitro stimulation, to minimise non-physiological conditions (Fig. 9). We formerly showed that IL-12, TNF, IFN-γ, CCL2, IL-10, IL-1α/β and IL-6 are upregulated in whole WNV-infected brains [1–3, 55]. Consistent with previously published work, Ly6C^hi^ macrophages and T cells were the principal source of NO and IFN-γ, respectively (Fig. 9a) [3, 55]. Ly6C^hi^ macrophages also had the highest expression of CD206 (Fig. 9a), confirming that macrophages can express both pro- and anti-inflammatory markers simultaneously. Microglia-like macrophages expressed NO and CD206 only marginally, suggesting a less inflammatory, alternative role for these cells. Of interest, was the primary production of IL-12/IL-23 p40 by microglia in the later phase of disease (Fig. 9a-h). Since IL-23 shares the p40 subunit with IL-12, we performed an ELISA and RNAse protection assay on total brain protein and RNA, respectively, to discriminate between these cytokines. The IL-23 (p19/p40) protein was below the limit of detection (Additional file 12), while marginal levels of IL-23 (p19) RNA were found in WNV-infected brains (Fig. 9d), indicating that IL-12 and not IL-23 was most likely to be produced by microglia. Notably, while the P2RY12^hi^CD86^+^ microglia subset had the highest frequency of IL-12/IL-23 p40^+^ cells (Fig. 9h), P2RY12^hi^CD86^−^ microglia, as the largest subset, produced most of the IL-12 (Fig. 9g). This suggests that these cells have role in T cell activation.

## Discussion

In the homeostatic CNS, multifarious gating strategies enable accurate identification of microglia by flow cytometry. However, under severe neuroinflammatory conditions, such as those induced by WNE, substantial numbers of infiltrating inflammatory monocytes adopt an activated microglial phenotype [1, 48], precluding the use of standard gating strategies. Accurately distinguishing microglia from infiltrating MDMs in the inflamed brain is required to determine their respective contribution to disease pathogenesis and/or recovery, potentially informing therapeutic approaches that target these cells. Here, we report for the first time, a simple, novel gating strategy to distinguish microglia from infiltrating myeloid cells under both homeostatic and extreme inflammatory conditions. This gating strategy minimises user gating bias and maximises accuracy of population analysis and sorting, and can be applied to a range of other neuroinflammatory models. Using this approach, we identified four consistent microglia subsets in the homeostatic and WNV-infected brain. These were P2RY12^hi^CD86^−^, P2RY12^hi^CD86^+^ and P2RY12^lo^CD86^−^ P2RY12^lo^CD86^+^. The four subsets identified in the homeostatic brain were phenotypically distinct from those in the infected brain, suggesting a change in their function and activity. Indeed, microglia adopted spatial and temporal disease specific signatures with increased neuronal infection. In stark contrast to the infiltrating myeloid population, microglia proliferated early in WNE, while late in disease they produced IL-12 and underwent apoptosis, indicating clear differential responses of each population.

To validate our gating strategy, we (1) blocked monocyte infiltration into the CNS, (2) adoptively transferred BM-derived monocytes and (3) stained blood leukocytes with an intravenous dye. These demonstrated that immigrant leukocytes did not populate the identified microglial gate. Conversely, depleting microglia with PLX5622 substantially diminished the cell numbers in this gate.

Various approaches have been developed to address the phenotypic convergence between microglia and MDM in disease; however, these have limited utility in our model. For example, double-heterozygous transgenic Ccr2rfp::Cx3cr1gfp mice, where GFP^+^ microglia are distinguishable from RFP^+^ monocytes [56], or Cx3cr1-cre-ER+/−tdTomatost/wt reporter mice, where long-lived CX3CR1^+^ microglia are tdTomato^+^ [48], have enabled separation of these cell types. However, CX3CR1 is also expressed by other myeloid cells, as well as memory T cells, B cells and NK cells [41], while macrophages in tamoxifen-treated Cx3cr1CreER mice are also tdTomato^+^ in the kidney, gut and CNS [40]. Other approaches have made use of the differential expression of CD44 [41, 42], VLA4 (CD49d) [43] and LFA1 (CD11a) to distinguish microglia from monocytes [44], but CD44 is also expressed by CNS resident macrophages and upregulated on microglia and CNS border-associated macrophages (BAMs) in EAE [57]. Finally, while recent ‘microglia-specific’ markers, TMEM119 [40] and P2RY12 [11], have enabled more accurate identification of microglia, both markers are downregulated during inflammatory neurodegeneration [51, 53, 58] and TMEM119 is also expressed by lymph node macrophages [59], potentially reducing their reliability for microglial identification [60].

In our model, microglia downregulated CX3CR1 over the course of infection, while Ly6C^hi^ macrophages expressed CX3CR1, with varied CCR2 expression. Microglial expression levels of CD44, LFA1 (CD11a) and VLA4 (CD49d) in WNE were similar to those seen on ‘microglia-like’ macrophages (Additional file 2). Moreover, microglia downregulated TMEM119, while infiltrating myeloid populations upregulated both P2RY12 and TMEM119 expression. Complicating this further, collagenase and DNase, used to maximise live leukocyte yields from CNS tissues, virtually abrogated TMEM119 labelling and significantly reduced CD44 labelling. Thus, previously published approaches cannot readily discriminate between microglia and infiltrating myeloid cells in our model, although 4D4, another nominal microglia-specific marker [61, 62], which was not used in this report, may be of value.

In WNE, infiltrating myeloid populations also expressed nominal border-associated macrophage markers, including CD206 and MHC-II [51, 63], hampering discrimination of infiltrating cells from border-associated macrophages. Thus, specific markers will be required to identify and evaluate their respective roles in WNE.

While we have not studied the detailed functions of the identified microglial subsets, kinetic analysis of cell surface expression strongly imply changes in function between these subsets over the course of disease. Indeed, significant increases in both P2RY12^lo^ microglial subsets occurred principally at the expense of the P2RY12^hi^ CD86^−^ subset, accompanied by a marked increase in BrdU incorporation in the latter, suggesting differentiation from the P2RY12^hi^ CD86^−^ subset with compensatory proliferation. Microglia with a higher expression of P2RY12 may be more important in responding to virus infection. In PRV infection, P2RY12 was required for microglial migration and phagocytosis of virus-infected neurons [24]. In contrast, this purinergic receptor may be less important in neurodegeneration, as it is consistently downregulated in AD, ALS and EAE, along with TMEM119 [51–53]. On the other hand, the two CD86^+^ microglial cell populations are likely to be involved in antigen presentation and T cell activation. A CD86^+^ population has also been identified in the CNS in both homeostasis and EAE [43]. Conversely, the CD86^−^ microglial subsets may have a role in chemotaxis, complement-mediated function and phagocytosis of iC3b or IgG/antigen complexes, based on their higher expression of CD11b, CD11c and CD64.

Over the course of infection, the total microglia population progressively downregulated CX3CR1, F4/80, TMEM119 and CD68, and progressively upregulated CD45. In contrast, expression of CD64, MerTK, CD86, MHC-II, CCR2 and Ly6C peaked at dpi 5 and 6, but decreased by dpi 7. Given the downregulation of these immunologically important markers, the role of microglia may be more critical early in disease, as suggested by Wheeler et al. [27]. Furthermore, the idea of a fixed set of ‘activation’ or homeostatic markers being up- or downregulated by microglia may not apply in all diseases. During infection, P2RY12^hi^ microglia had the highest simultaneous expression of both activation and homeostatic markers. Thus, the expression of individual markers is likely to be disease- and stage-specific, together constituting a unique disease signature.

Here, we also show the upregulation of ‘microglia-specific’ markers on infiltrating Ly6C^hi^ and microglia-like macrophages, in particular, in the rostral parts of the brain. Similarly, in two stroke models, macrophages infiltrating the CNS [64] or ectopically placed in peri-infarct areas [65], became TMEM119^+^ and/or P2RY12^+^ and/or *Sall1*^*+*^. The prolonged time spent in the CNS, may induce infiltrating cells to become more microglia-like, since the unique brain microenvironment shapes the identity of microglia [13]. Microglia-like macrophages may represent a distinct subset arising from BM during infection, although they are likely to differentiate from Ly6C^hi^ infiltrating macrophages, because their numbers are proportionately reduced when immigration of Ly6C^hi^ monocytes is blocked with monoclonal antibodies. The microglia-like macrophage population in WNE brains also had the greatest population of apoptotic and dead cells. Thus, once Ly6C^hi^ macrophages enter the brain, they may acquire a microglia-like phenotype and eventually die after performing effector functions.

Interestingly, at dpi 5 microglia showed the greatest incorporation of BrdU. Their reduced proliferative capacity from dpi 6 onwards was accompanied by an increase in microglial cell death. It has previously been suggested that flaviviral infection does not promote microglial proliferation [33, 34] and that increased numbers of ‘microglia’ during WNE were due to the infiltration of BM-derived cells [1]. Our study emphasises the importance both of accurate microglial cell identification as well as detailed examination of kinetic changes during infection. Microglial cell proliferation has been well documented in JEV [66], TMEV [60, 67] and VSV infection [31], as well as in EAE [68]. Furthermore, similar to our findings, the frequency of proliferating microglia in EAE increased then decreased over the course of disease, with TUNNEL^+^ apoptotic microglia detected in the later phases [43, 51]. Microglial proliferation and apoptosis is tightly coupled [9]. We hypothesise that microglia proliferate early in infection to enhance the response against WNV, but further proliferation becomes redundant as increasing numbers of MDMs enter the brain parenchyma. The increased number of dying microglia in WNE brains presumably enable these cells to return to homeostatic numbers. This may also apply in EAE.

We found that microglia were responsible for the primary expression of IL-12 in the later phase of infection. Similarly, in TMEV-infection, microglia exclusively expressed *Il12b* and *Il12rb1* [60]. A previous report showed TLR7-IL-23-dependent homing of peripheral immune cells to the brain in WNV-infected mice [69]. In contrast, we found limited IL-23. This could be due to differences in virus strain or inoculation routes.

The precise functions subserved by microglia and MDM subsets identified here remain to be fully determined. IL-12 production supports a role for microglia in enhancing NK and T cell responses to aid viral clearance. Consistent with this, recent studies showed ineffective CD4^+^ T cell [27] or CD8^+^ T cell [29] responses in viral infection following microglial depletion with PLX5622. However, it must be noted that, as well as causing mass microglial death, requiring yet undefined processes to clear these cells, PLX5622 also modulates other myeloid cells dependent on CSF1R signalling [30]. Thus, the non-physiological conditions imposed by PLX5622 may perturb normal immune responses, making it difficult to define the contributions of microglia to disease. This emphasises the need to examine these cells without depletion. Using intravital imaging, Moseman et al. [32] confirmed the involvement of microglia to an effective T cell response in VSV encephalitis. Microglia were required for cross-presentation to CD8^+^ T cells, to contain and prevent the fatal spread of VSV [32]. In WNE, since microglia produce IL-12 in the later phase of disease, production could also be a response to T cells producing large quantities of IFN-γ at dpi 7 [3], as IFN-γ can stimulate the production of either IL-12 subunit [70].

## Conclusion

Historically, the convergence of resident and infiltrating myeloid cell phenotypes in the inflamed brain has hindered our understanding of the function of microglia in disease. While new investigative tools have improved population resolution and analyses of these cells, these are unreliable in the inflamed brain. Microglia-specific markers were downregulated by microglia and/or expressed de novo by peripherally derived cells in WNV-infection, while microglial cell depletion with PLX5622 can target peripheral cells and create non-physiological conditions in the brain. Here, we devised a simple gating strategy to identify and track microglia during severe inflammatory changes in the CNS, while maintaining the natural immune status of the animal. Using this approach, we showed that resident microglia undergo unique temporal- and spatial- specific alterations from MDMs. This approach can be applied to other neuroinflammatory models to understand the contribution of microglia to disease, as well as to inform therapeutics that can specifically target and modulate these cells in WNE.

## Supplementary Information

**Additional file 1 **Collagenase and DNase digestion increases live leukocyte yield from murine brains. Percent **(a, c, e, g, I, k)** and number **(b, d, f, h, j, l)** of total live **(a, b, g, h)**, live CD45^+^CD11b^+^, CD45^+^CD11b^-^ **(c, d, I, j)** and live myeloid and lymphoid populations **(e, f, k, l)** from mock-infected **(a-f)** and WNV-infected **(g-l)** murine brains. **a, b, g, h,** *P<0.0332, Mann-Whitney Test, **c-f, i-l**, *P<0.0332, **P<0.0021, ***P<0.0002, Two -way ANOVA with a Šídák's multiple comparisons test.

**Additional file 2 **Overlapping expression of 'infiltrating macrophage' markers on resident and infiltrating myeloid populations in WNV-infected brains. **a-e** Histograms and FACs plots showing the expression of CD44 **(a)**, LFA1 (CD11a) **(b)**, VLA4 (CD49d) **(c)**, Ly6C **(d)** and CCR2 **(e)** on microglia from homeostatic (blue) and infected (green) brains relative to infiltrating microglia-like (orange) and Ly6C^hi^ macrophages (dark pink) in WNV dpi 7 brains.

**Additional file 3 **Microglia are easily identifiable in the homoeostatic brain. **a-d** Quality control gates, including time **(a)**, single cells **(b)**, non-debris **(c)** and live cell **(d)** gates were applied before analysing cells. Neutrophils were also excluded by their expression of Ly6G **(e)**. **f** Gating strategy one identifies a ‘resting’ microglia population (CD45^lo^ CD11b^+^) (R1: blue) and an ‘activated’ microglia population (CD45^int^ CD11b^+^) (R2: red). **h, j** Gating strategy two, does not use ‘microglia-specific markers’ and identifies microglia as CX3CR1^+^ CD45^lo-int^ CD11b^+^ Ly6C^-/lo^. Gating strategy three is a revised gating strategy which identifies microglia as CX3CR1^+^ CD45^lo-int^ CD11b^+^ P2RY12^+^ Ly6C^-/lo^ (**h, I, l**). **n, o** Gating strategy four uses a limited number of markers and identifies microglia as CD45^lo-int^ P2RY12^+^CD11b^+^CX3CR1^+^. **g, k, m, p** ‘Microglia’ populations gated using strategies 1 **(g)**, 2 **(k)**, 3 **(m)**, and 4 **(p)**, overlaid onto a tSNE plot, clustered on live cells from mock-infected brains. **q, r** Number **(q)** and frequency **(r)** of ‘microglia’ gated using strategies 1-4. Data is presented as mean ± SEM, from two independent experiments with at least six mice per group. **P<0.0021, Kruskal-Wallis test and Dunn’s multiple comparisons test.

**Additional file 4** Gating strategy used to identify resident microglia and infiltrating myeloid and lymphoid populations in the inflamed brain.

**Additional file 5** Immune profiles of resident microglia and infiltrating myeloid cells in WNE at dpi 7.

**Additional file 6 **TMEM119 and CD44 are masked by collagenase and DNase brain digestion. **a-f** tSNE plot clustered on myeloid cells from mock-infected and WNV dpi 7 brains digested with and without collagenase/DNase. tSNE plot representing enzyme and non-enzyme digested brain cells from mock- **(a)** and WNV-infected **(b)** animals clustered on live, CD11b^+^ and CD45^+^ cells. Annotation of myeloid cells on tSNE plots representing mock-infected **(c, e)** and WNV-infected brains **(d, f)** with **(c, d)** and without **(e, f)** enzyme digestion. **g-l** Histograms showing the loss of TMEM119 on microglia in mock-infected brains **(g)** and CD44 on infiltrating Ly6C^hi^ macrophages and neutrophils in WNV-infected **(j)**, enzyme-digested brains. tSNE plots showing the reduced expression of TMEM119 **(h, i)** and CD44 **(k, l)** in collagenase and DNase processed **(h, k)**, mock-infected **(h)** and WNV-infected **(k)** brains, respectively. Data is representative of at least two independent experiments, with a minimum 6 animals per group.

**Additional file 7** Four microglia phenotypes identified in the homeostatic and infected brain have differential immune profiles. Histograms showing the expression of selected markers on/in P2RY12^hi^CD86^+^ (dark purple), P2RY12^lo^CD86^-^ (light green), P2RY12^lo^CD86^+^ (light purple) and P2RY12^hi^CD86^-^ (dark green) microglia at dpi 0 (blue) and dpi 7 (orange).

**Additional file 8 **‘Microglia-specific’ marker TMEM119 is expressed by infiltrating macrophages populations in the brain but not in the bone marrow or blood. **a, b** Histograms showing the expression of TMEM119 on microglia **(a)** in naïve and WNV-infected mice and on microglia, Ly6C^hi^ macrophages and microglia-like macrophages **(b)** in WNV dpi 7 brains. Fluorescence minus one (FMO) and single stains (ss) for TMEM119 are shown each plot. **c-f** tSNE plot clustered on live cells from naïve and WNV dpi 7 brains. **c, d** Annotation of populations found in the mock-infected **(c)** and infected **(d)** brain tSNE. **e, f** Relative expression TMEM119 on tSNE plots representing naïve **(e)** and WNV-dpi 7 **(f)** brains. **g** Heatmap showing the expression TMEM119 on neutrophils, microglia, microglia-like macrophages and Ly6Chi macrophages from four brain samples (1-4). **h** Expression of TMEM119 on peripheral bone marrow and blood Ly6C^hi^ monocytes and Ly6C^hi^ macrophages in WNV dpi 7 brains. **i-k** tSNE plot clustered on live, SSC-A^lo^ and Ly6G^+/-^ bone marrow, live, SSC-A^lo^ and Ly6G^+/-^ blood cells and live brain cells from WNV dpi 7 brains. Data is representative of at least two independent experiments, with a minimum 7 animals per group.

**Additional file 9 **‘Microglia-specific’ marker P2RY12 is expressed by infiltrating macrophages populations in the brain but not in the bone marrow or blood. **a, b** Histograms showing the expression of P2RY12 on microglia **(a)** in naïve and WNV-infected mice and on microglia, Ly6C^hi^ macrophages and microglia-like macrophages **(b)** in WNV dpi 7 brains. Fluorescence minus one (FMO) and single stains (ss) for P2RY12 are shown each plot. **c-f** tSNE plot clustered on live cells from naïve and WNV dpi 7 brains. **c, d** Annotation of populations found in the mock-infected **(c)** and infected **(d)** brain tSNE. **e, f** Relative expression P2RY12 on tSNE plots representing naïve **(e)** and WNV-dpi 7 **(f)** brains. **g** Heatmap showing the expression P2RY12 on neutrophils, microglia, microglia-like macrophages and Ly6Chi macrophages from four brain samples (1-4). **h** Expression of P2RY12 on peripheral bone marrow and blood Ly6C^hi^ monocytes and Ly6C^hi^ macrophages in WNV dpi 7 brains. **i-k** tSNE plot clustered on live, SSC-A^lo^ and Ly6G^+/-^ bone marrow, live, SSC-A^lo^ and Ly6G^+/-^ blood cells and live brain cells from WNV dpi 7 brains. Data is representative of at least two independent experiments, with a minimum 7 animals per group.

**Additional file 10 **Brain cells stained with and without cyanine dye blocker and 4 commercially available CD86 conjugates. **a-c** FACs plots showing murine brain cells from dpi 0 **(a)**, 5 **(b)** and 7 **(c)** stained with CD86 conjugated to BV605, FITC, PE/Cy7 and APC/Cy7, with and without True-stain monocyte blocker^TM^ (Biolegend). **d** Percent of CD86^+^ microglia from dpi 0, 5 and 7 brains stained with CD86 conjugated to BV605, FITC, PE/Cy7 and APC/Cy7 with and without True-stain monocyte blocker^TM^. Fluorescence minus one (FMO) stained with and without True-stain monocyte blocker^TM^ (Biolegend) is shown for CD86.

**Additional file 11** Temporal immunophenotypic changes in microglial cell populations during WNE. Histograms showing the expression of selected markers on/in P2RY12^hi^CD86^+^, P2RY12^lo^CD86^-^, P2RY12^lo^CD86^+^ and P2RY12^hi^CD86^-^ microglia at dpi 0 (purple), 4 (orange), 5 (green), 6 (pink) and 7 (blue). Data is representative of at least two independent experiments.

**Additional file 12** IL-23 is under the detection threshold in WNV infected brains. Quantity of IL-23 (p19/p40) in mock and WNV-infected mice at dpi 7, as determined using an ELISA.

## Data Availability

The datasets used and/or analysed during the current study are available from the corresponding author on reasonable request.

## Abbreviations

AD: Alzheimer’s disease
ALS: Amyotrophic lateral sclerosis
BM: Bone marrow
CNS: Central nervous system
dpi: day post infection
EAE: Experimental autoimmune encephalomyelitis
FMO: Fluorescence minus one
JEV: Japanese encephalitis virus
LD_100_: Lethal dose 100%
MDM: Monocyte-derived macrophages
MFI: Median fluorescence intensity
MHV: Murine hepatitis virus
PFU: Plaque forming units
PRV: Pseudorabies virus
TMEV: Theiler’s encephalomyelitis virus
tSNE: t-Distributed Stochastic Neighbor Embedding
UVLD: UV-excitable LIVE/DEAD
VSV: Vesicular stomatitis virus
WNE: West Nile virus encephalitis
WNV: West Nile virus
