# Supplementary figures and images for: High-parameter cytometry unmasks microglial cell spatio-temporal response kinetics in severe neuroinflammatory disease
Source: J Neuroinflammation. 2021 Jul 26;18:166. doi: 10.1186/s12974-021-02214-y (PMC8314570; doi:10.1186/s12974-021-02214-y)

# Mock-infected

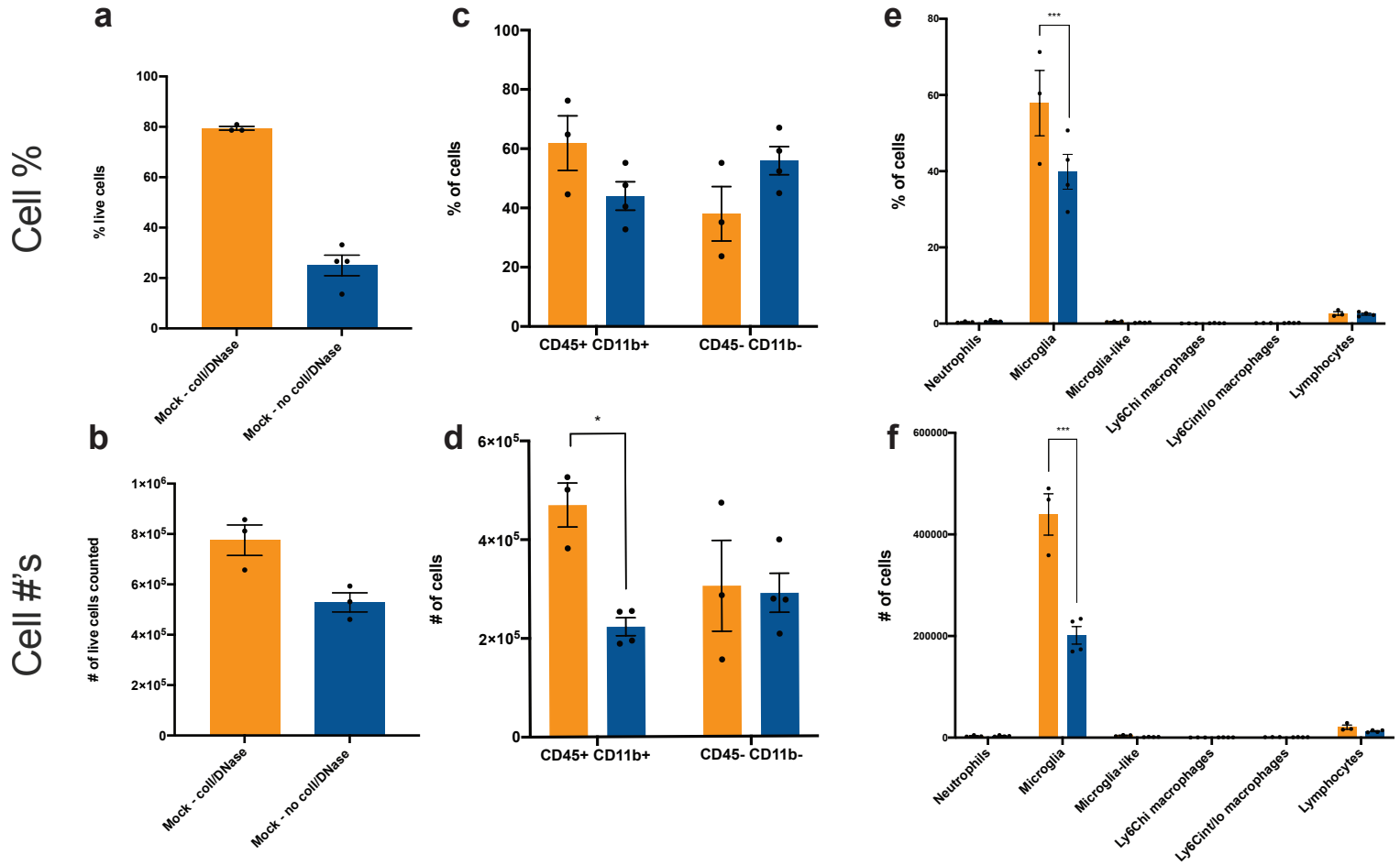

# WNV-infected

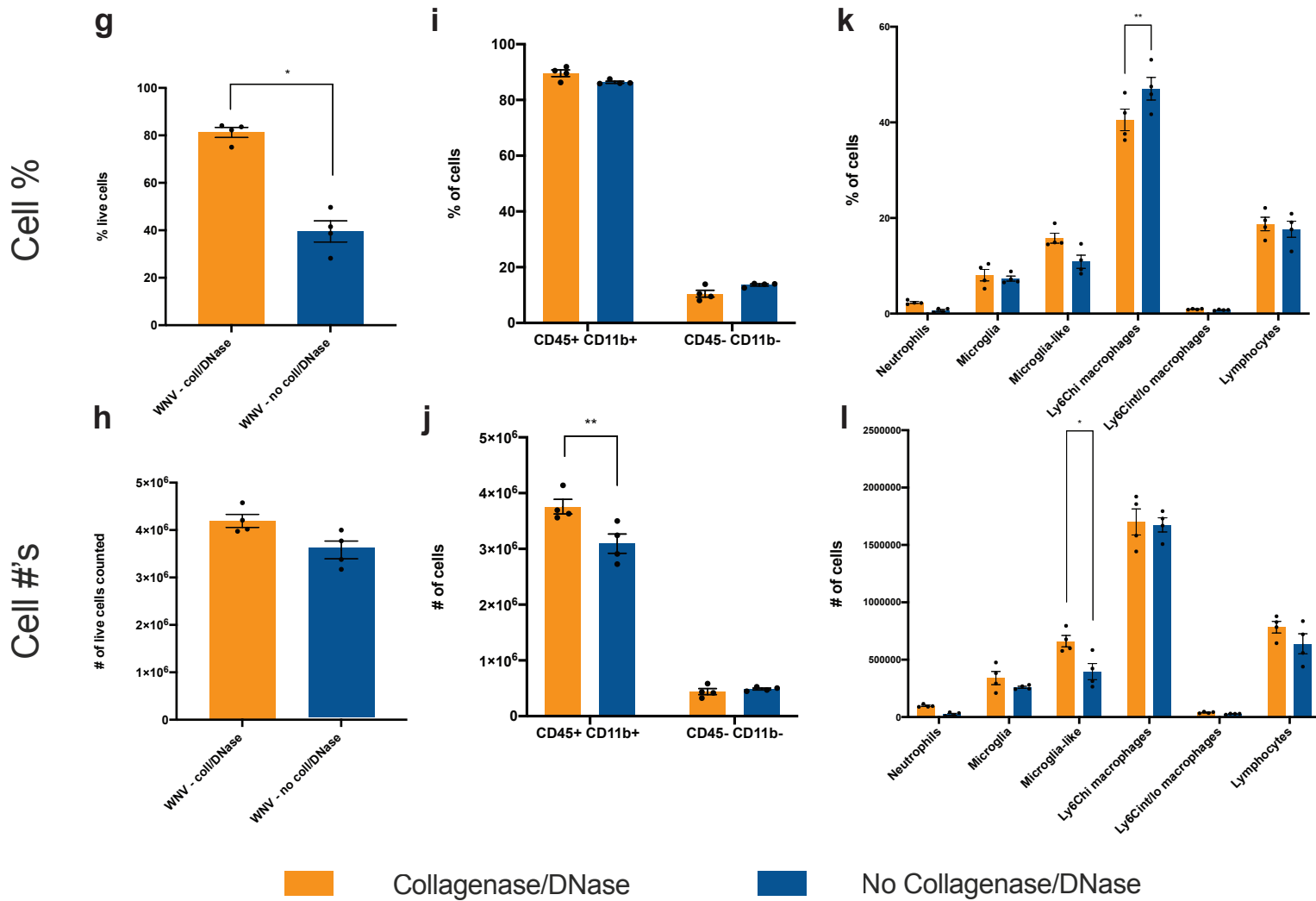

Supplement: Supplementary file 1 — Additional file 1 Collagenase and DNase digestion increases live leukocyte yield from murine brains. Percent (a, c, e, g, I, k) and number (b, d, f, h, j, l) of total live (a, b, g, h), live CD45+CD11b+, CD45+CD11b- (c, d, I, j) and live myeloid and lymphoid populations (e, f, k, l) from mock-infected (a-f) and WNV-infected (g-l) murine brains. a, b, g, h, *P<0.0332, Mann-Whitney Test, c-f, i-l, *P<0.0332, **P<0.0021, ***P<0.0002, Two -way ANOVA with a Šídák's multiple comparisons test. [file 12974_2021_2214_MOESM1_ESM.pdf]

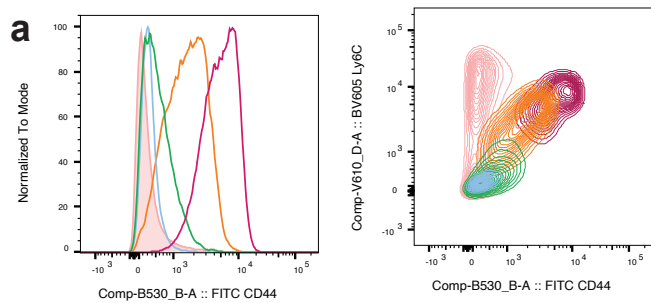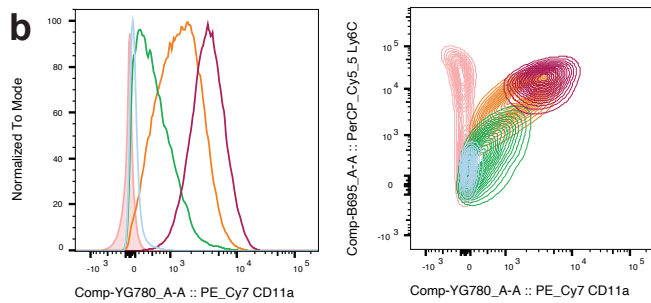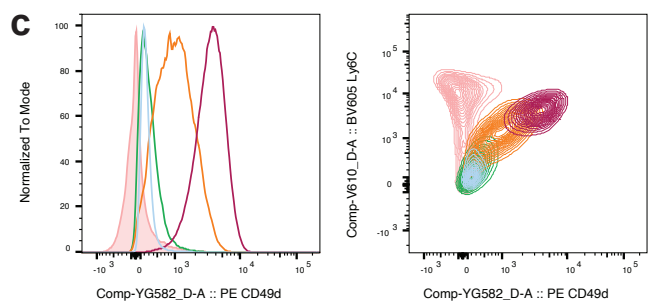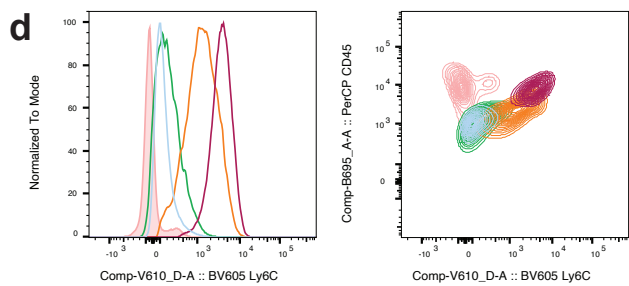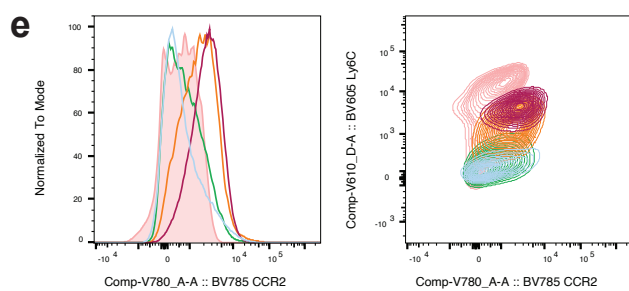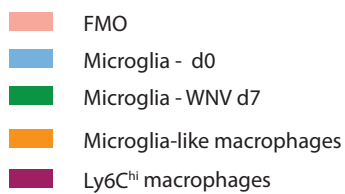

Supplement: Supplementary file 2 — Additional file 2 Overlapping expression of 'infiltrating macrophage' markers on resident and infiltrating myeloid populations in WNV-infected brains. a-e Histograms and FACs plots showing the expression of CD44 (a), LFA1 (CD11a) (b), VLA4 (CD49d) (c), Ly6C (d) and CCR2 (e) on microglia from homeostatic (blue) and infected (green) brains relative to infiltrating microglia-like (orange) and Ly6Chi macrophages (dark pink) in WNV dpi 7 brains. [file 12974_2021_2214_MOESM2_ESM.pdf]

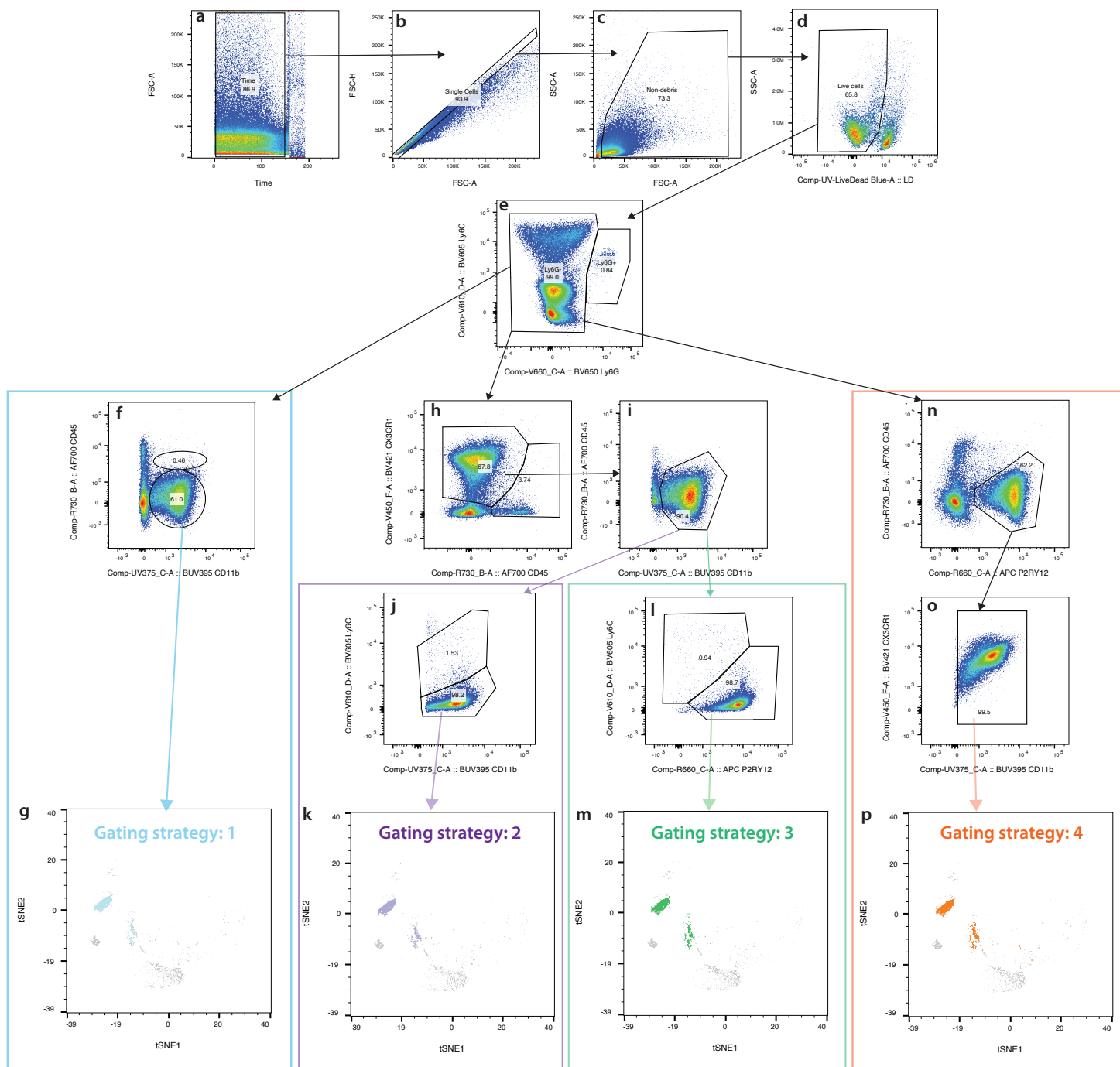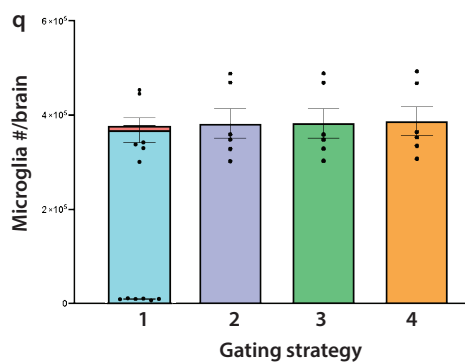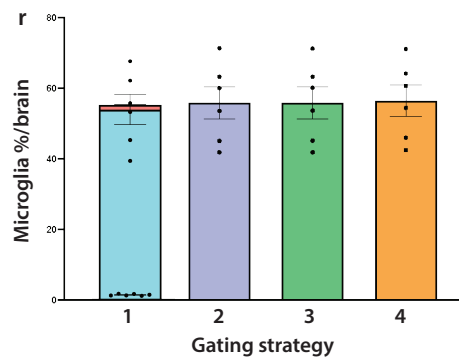

Supplement: Supplementary file 3 — Additional file 3 Microglia are easily identifiable in the homoeostatic brain. a-d Quality control gates, including time (a), single cells (b), non-debris (c) and live cell (d) gates were applied before analysing cells. Neutrophils were also excluded by their expression of Ly6G (e). f Gating strategy one identifies a ‘resting’ microglia population (CD45lo CD11b+) (R1: blue) and an ‘activated’ microglia population (CD45int CD11b+) (R2: red). h, j Gating strategy two, does not use ‘microglia-specific markers’ and identifies microglia as CX3CR1+ CD45lo-int CD11b+ Ly6C-/lo. Gating strategy three is a revised gating strategy which identifies microglia as CX3CR1+ CD45lo-int CD11b+ P2RY12+ Ly6C-/lo (h, I, l). n, o Gating strategy four uses a limited number of markers and identifies microglia as CD45lo-int P2RY12+CD11b+CX3CR1+. g, k, m, p ‘Microglia’ populations gated using strategies 1 (g), 2 (k), 3 (m), and 4 (p), overlaid onto a tSNE plot, clustered on live cells from mock-infected brains. q, r Number (q) and frequency (r) of ‘microglia’ gated using strategies 1-4. Data is presented as mean ± SEM, from two independent experiments with at least six mice per group. **P<0.0021, Kruskal-Wallis test and Dunn’s multiple comparisons test. [file 12974_2021_2214_MOESM3_ESM.pdf]

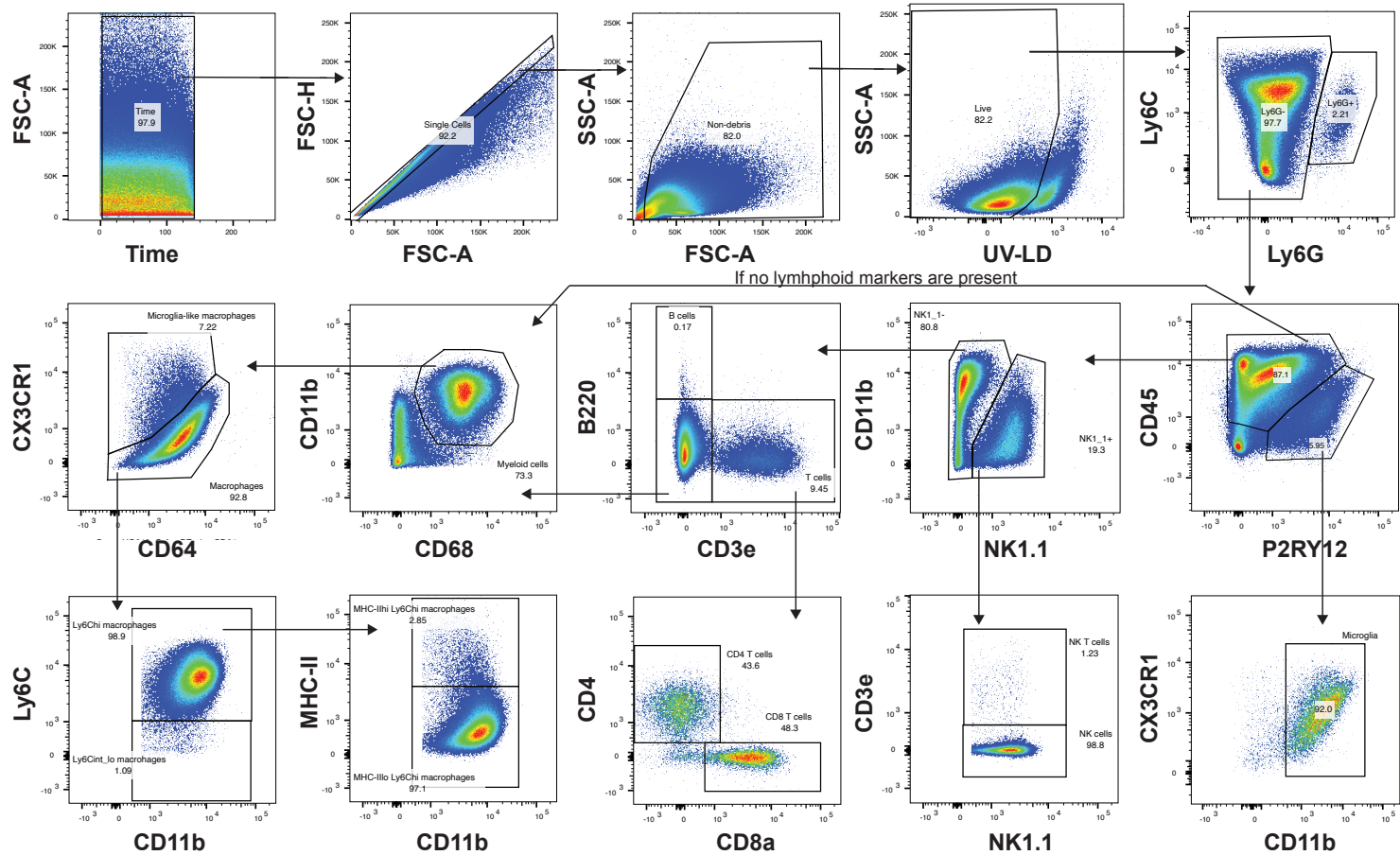

Supplement: Supplementary file 4 — Additional file 4 Gating strategy used to identify resident microglia and infiltrating myeloid and lymphoid populations in the inflamed brain. [file 12974_2021_2214_MOESM4_ESM.pdf]

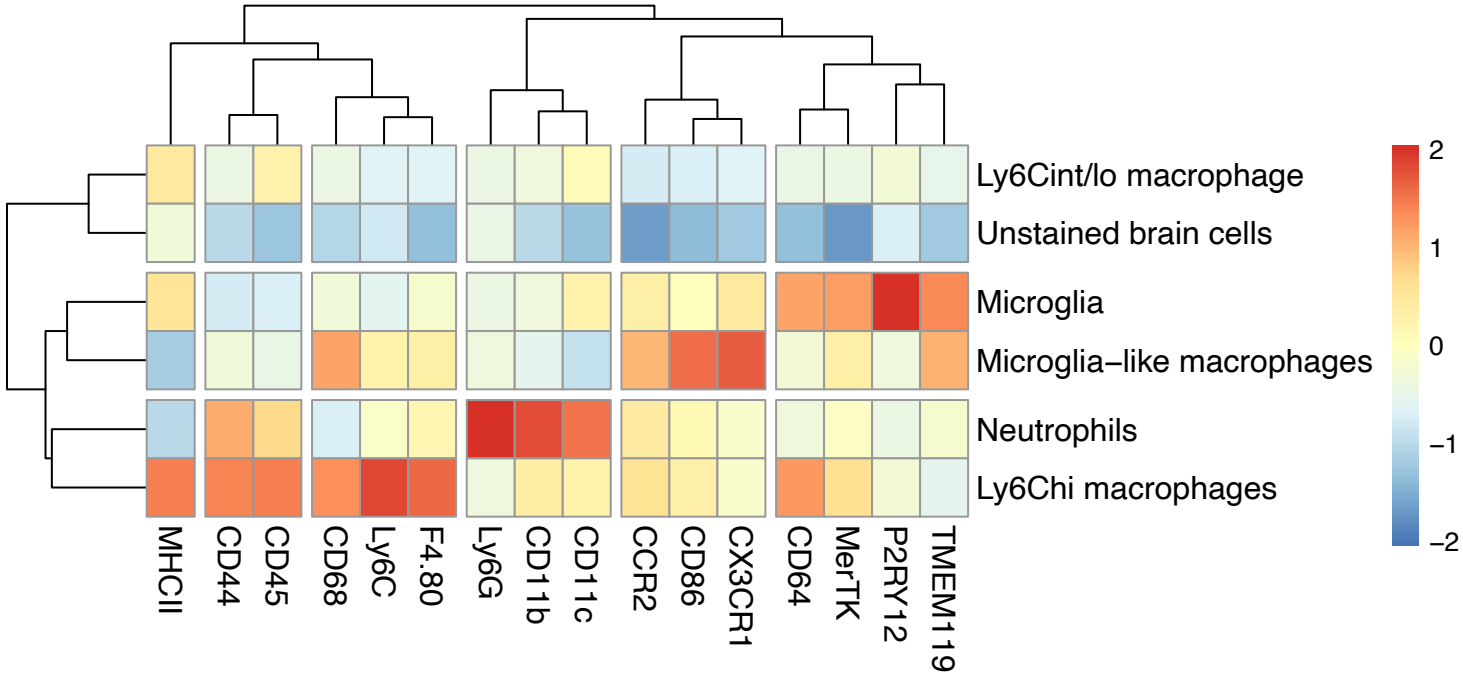

Supplement: Supplementary file 5 — Additional file 5 Immune profiles of resident microglia and infiltrating myeloid cells in WNE at dpi 7. [file 12974_2021_2214_MOESM5_ESM.pdf]

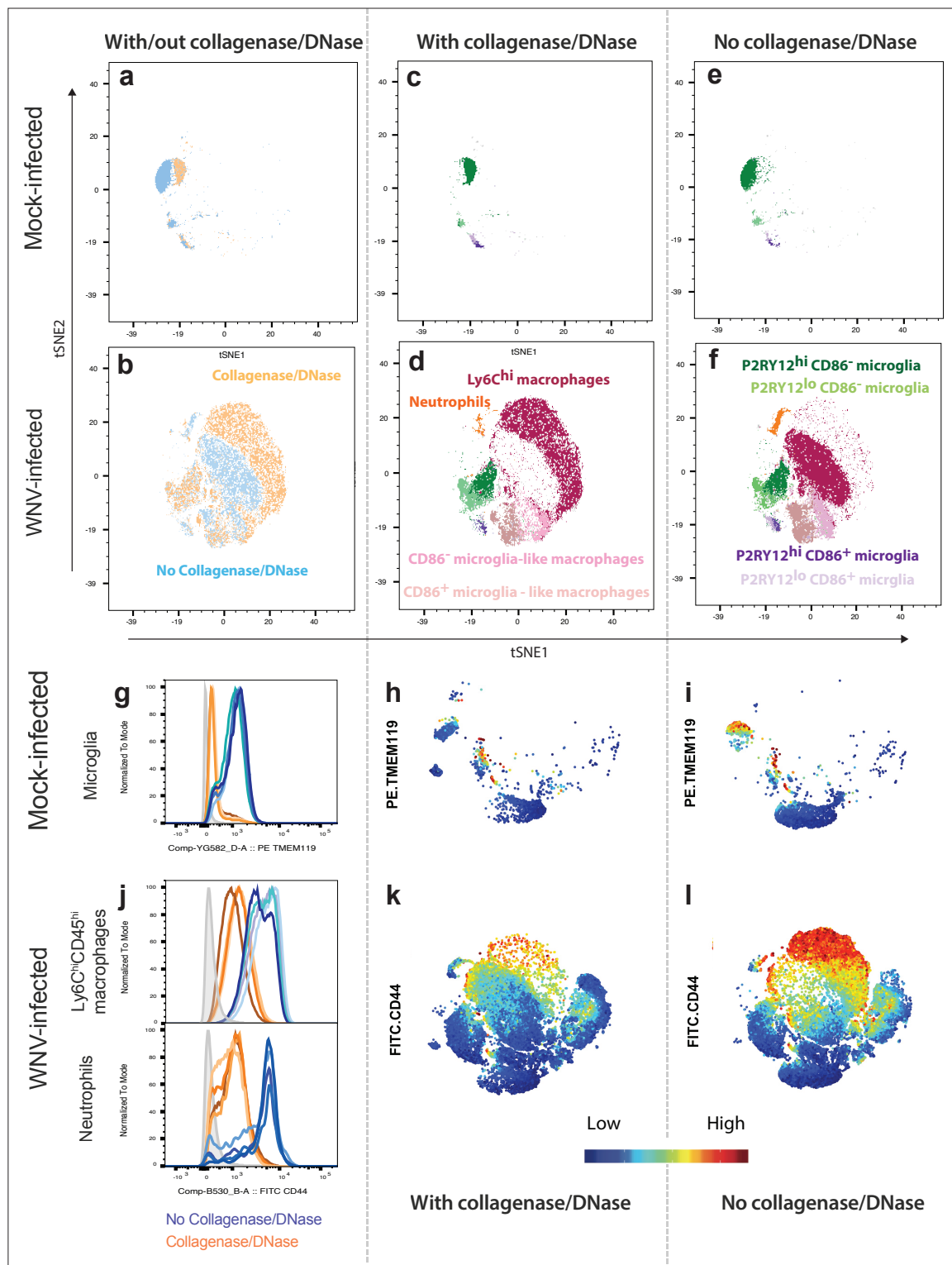

Supplement: Supplementary file 6 — Additional file 6 TMEM119 and CD44 are masked by collagenase and DNase brain digestion. a-f tSNE plot clustered on myeloid cells from mock-infected and WNV dpi 7 brains digested with and without collagenase/DNase. tSNE plot representing enzyme and non-enzyme digested brain cells from mock- (a) and WNV-infected (b) animals clustered on live, CD11b+ and CD45+ cells. Annotation of myeloid cells on tSNE plots representing mock-infected (c, e) and WNV-infected brains (d, f) with (c, d) and without (e, f) enzyme digestion. g-l Histograms showing the loss of TMEM119 on microglia in mock-infected brains (g) and CD44 on infiltrating Ly6Chi macrophages and neutrophils in WNV-infected (j), enzyme-digested brains. tSNE plots showing the reduced expression of TMEM119 (h, i) and CD44 (k, l) in collagenase and DNase processed (h, k), mock-infected (h) and WNV-infected (k) brains, respectively. Data is representative of at least two independent experiments, with a minimum 6 animals per group. [file 12974_2021_2214_MOESM6_ESM.pdf]

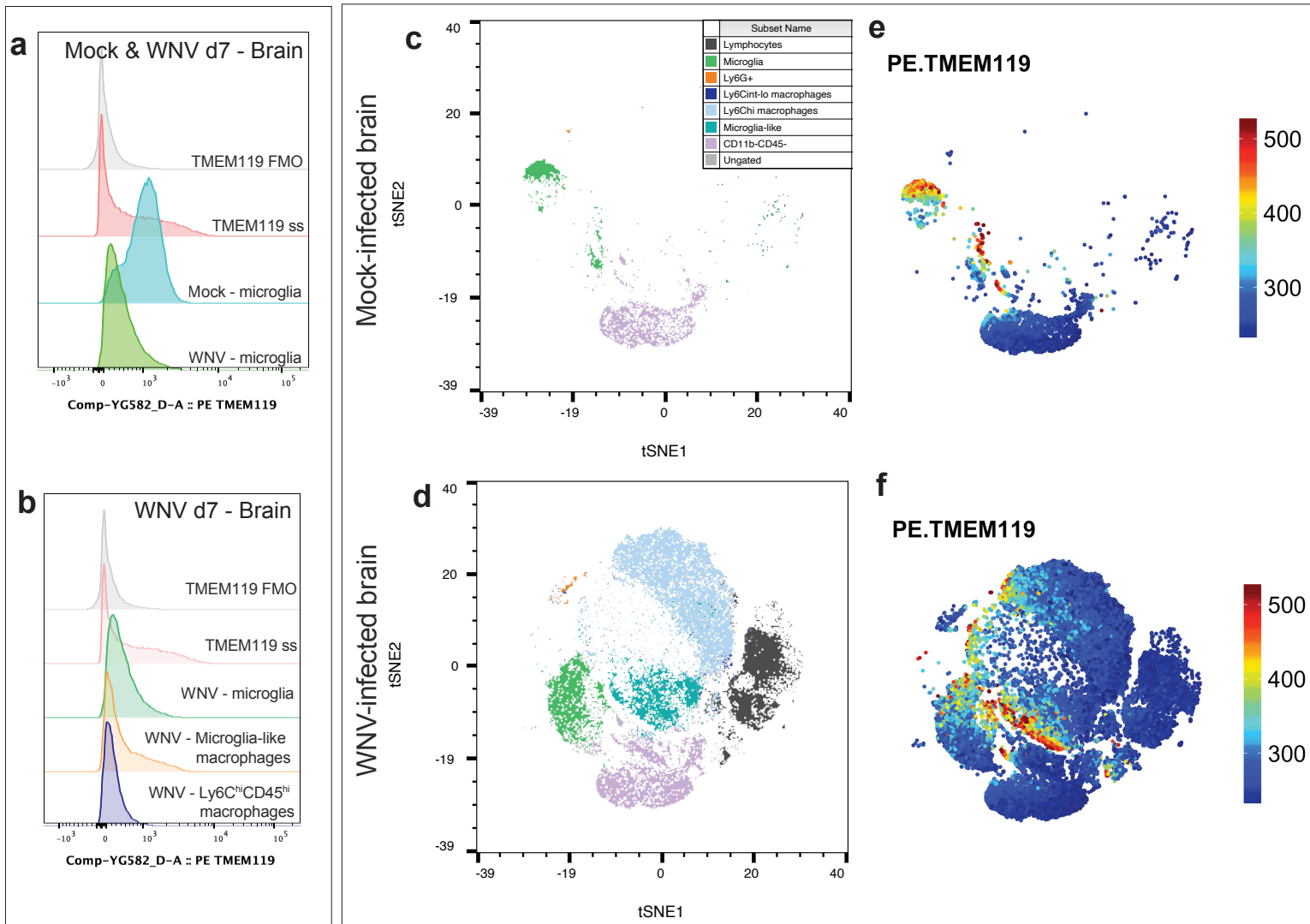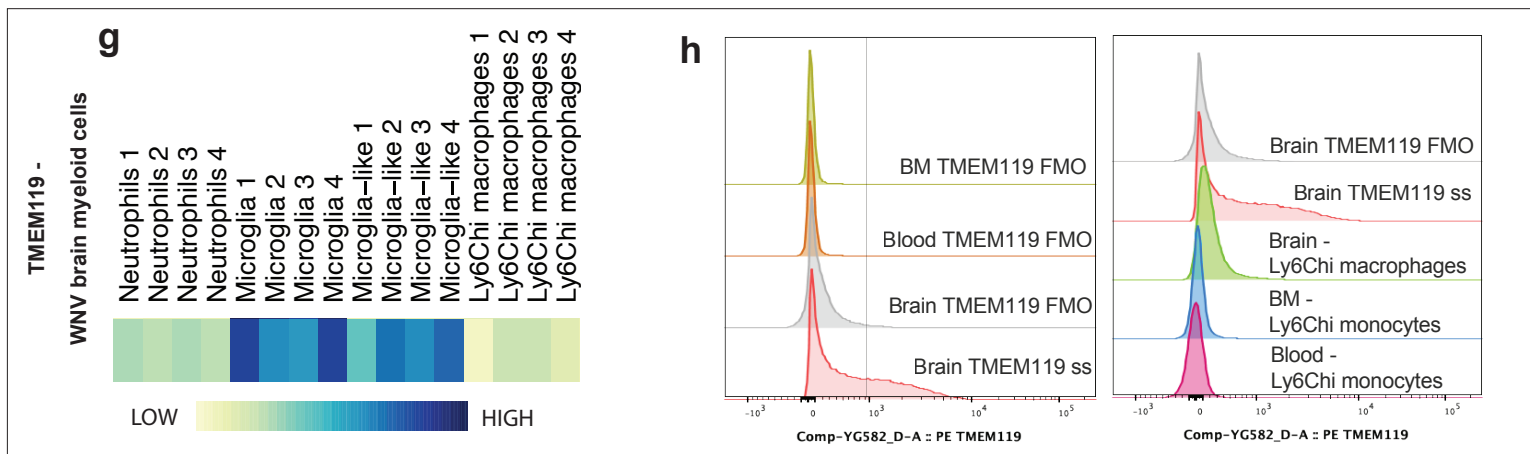

Supplement: Supplementary file 8 — Additional file 8 ‘Microglia-specific’ marker TMEM119 is expressed by infiltrating macrophages populations in the brain but not in the bone marrow or blood. a, b Histograms showing the expression of TMEM119 on microglia (a) in naïve and WNV-infected mice and on microglia, Ly6Chi macrophages and microglia-like macrophages (b) in WNV dpi 7 brains. Fluorescence minus one (FMO) and single stains (ss) for TMEM119 are shown each plot. c-f tSNE plot clustered on live cells from naïve and WNV dpi 7 brains. c, d Annotation of populations found in the mock-infected (c) and infected (d) brain tSNE. e, f Relative expression TMEM119 on tSNE plots representing naïve (e) and WNV-dpi 7 (f) brains. g Heatmap showing the expression TMEM119 on neutrophils, microglia, microglia-like macrophages and Ly6Chi macrophages from four brain samples (1-4). h Expression of TMEM119 on peripheral bone marrow and blood Ly6Chi monocytes and Ly6Chi macrophages in WNV dpi 7 brains. i-k tSNE plot clustered on live, SSC-Alo and Ly6G+/- bone marrow, live, SSC-Alo and Ly6G+/- blood cells and live brain cells from WNV dpi 7 brains. Data is representative of at least two independent experiments, with a minimum 7 animals per group. [file 12974_2021_2214_MOESM8_ESM.pdf]

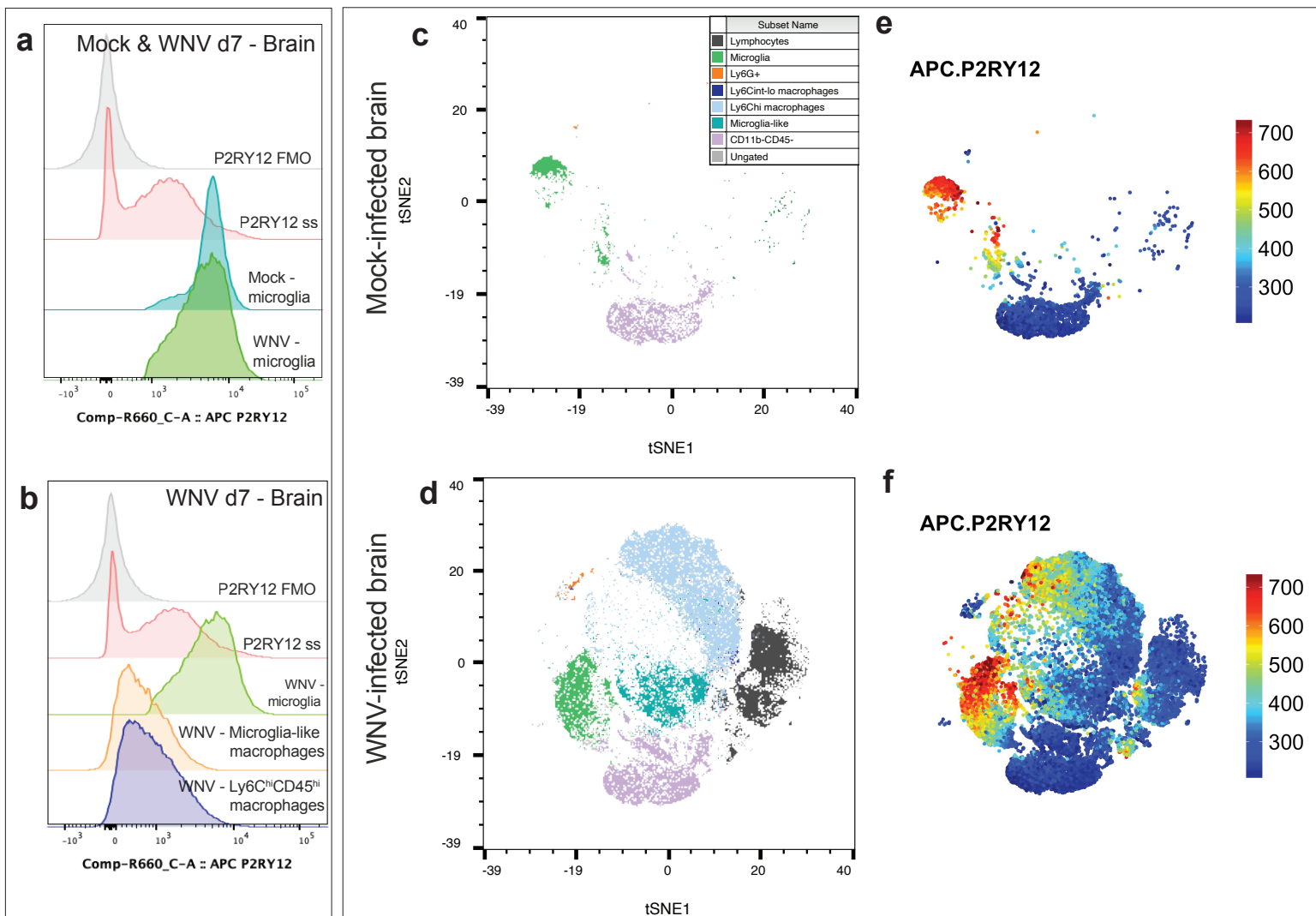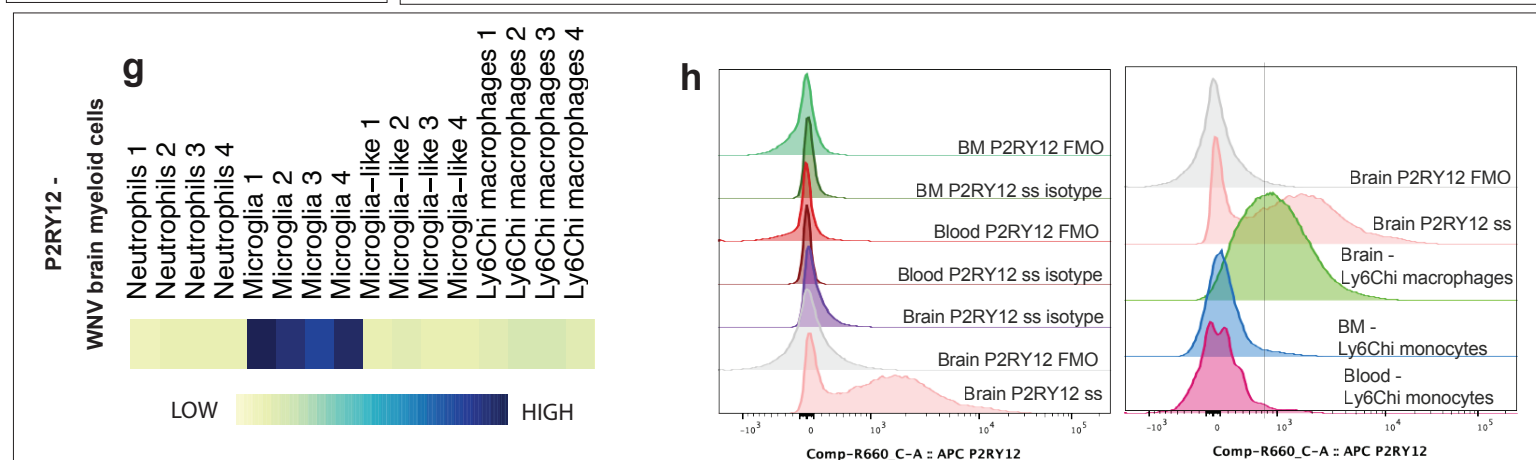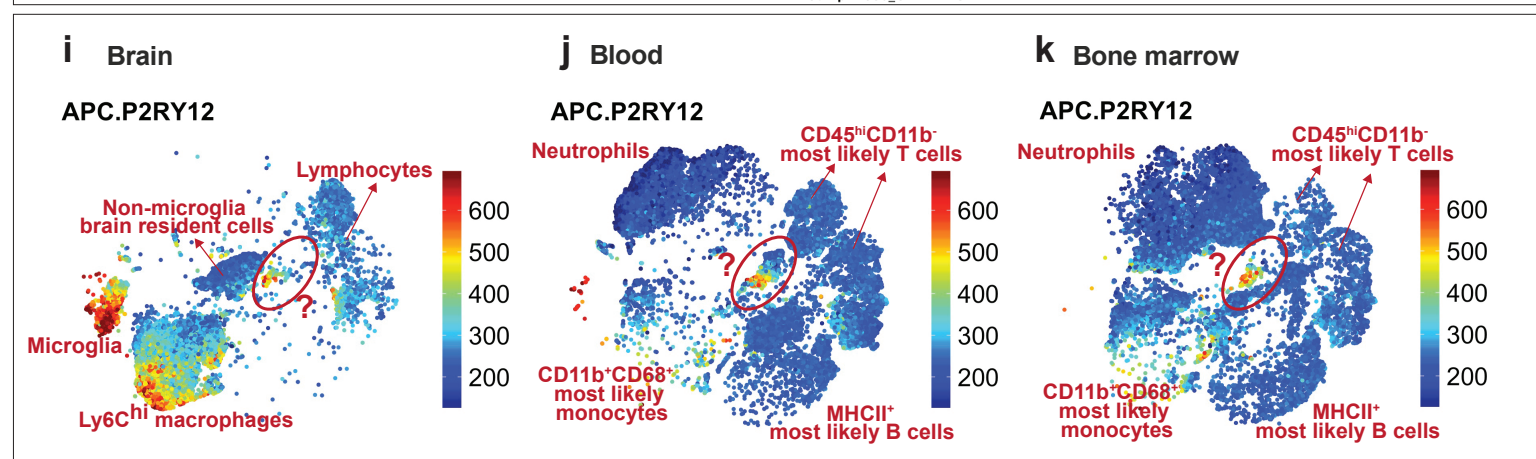

Supplement: Supplementary file 9 — Additional file 9 ‘Microglia-specific’ marker P2RY12 is expressed by infiltrating macrophages populations in the brain but not in the bone marrow or blood. a, b Histograms showing the expression of P2RY12 on microglia (a) in naïve and WNV-infected mice and on microglia, Ly6Chi macrophages and microglia-like macrophages (b) in WNV dpi 7 brains. Fluorescence minus one (FMO) and single stains (ss) for P2RY12 are shown each plot. c-f tSNE plot clustered on live cells from naïve and WNV dpi 7 brains. c, d Annotation of populations found in the mock-infected (c) and infected (d) brain tSNE. e, f Relative expression P2RY12 on tSNE plots representing naïve (e) and WNV-dpi 7 (f) brains. g Heatmap showing the expression P2RY12 on neutrophils, microglia, microglia-like macrophages and Ly6Chi macrophages from four brain samples (1-4). h Expression of P2RY12 on peripheral bone marrow and blood Ly6Chi monocytes and Ly6Chi macrophages in WNV dpi 7 brains. i-k tSNE plot clustered on live, SSC-Alo and Ly6G+/- bone marrow, live, SSC-Alo and Ly6G+/- blood cells and live brain cells from WNV dpi 7 brains. Data is representative of at least two independent experiments, with a minimum 7 animals per group. [file 12974_2021_2214_MOESM9_ESM.pdf]

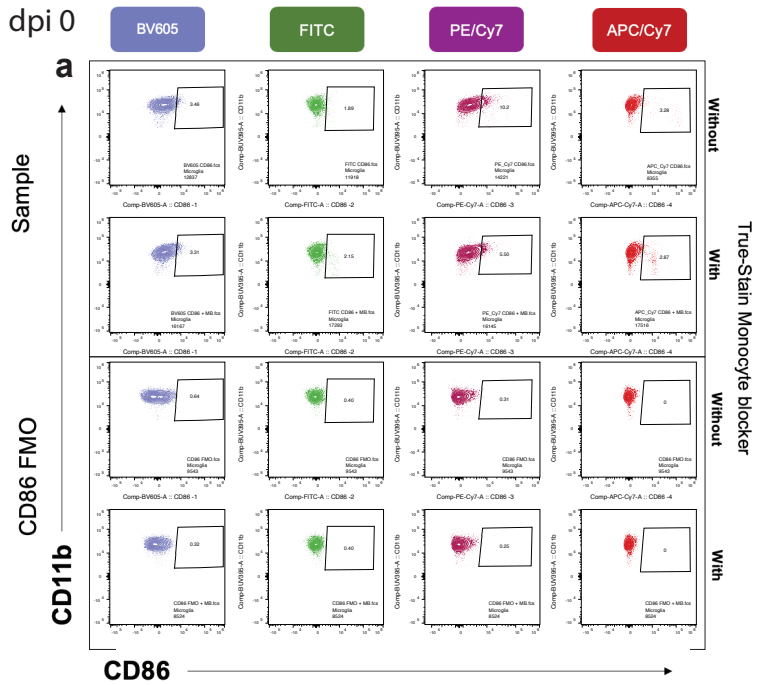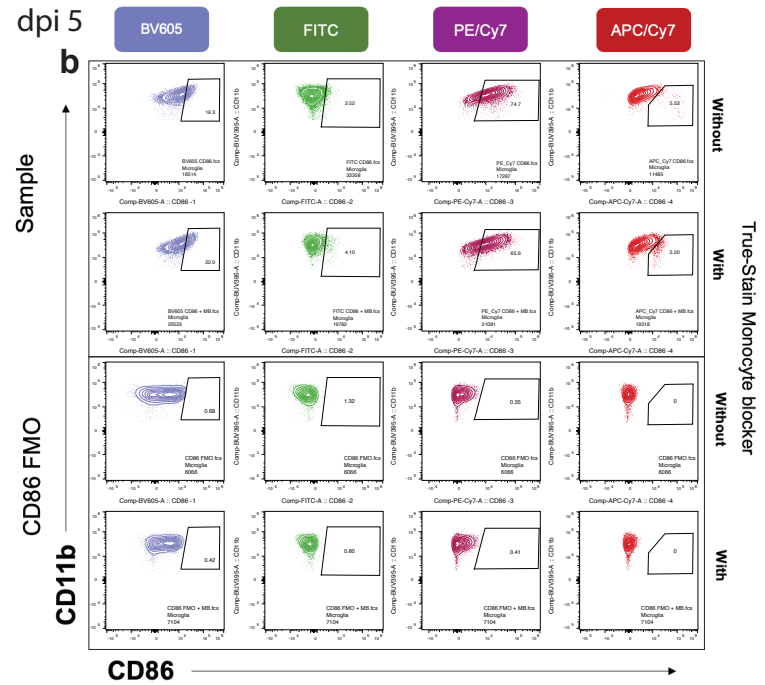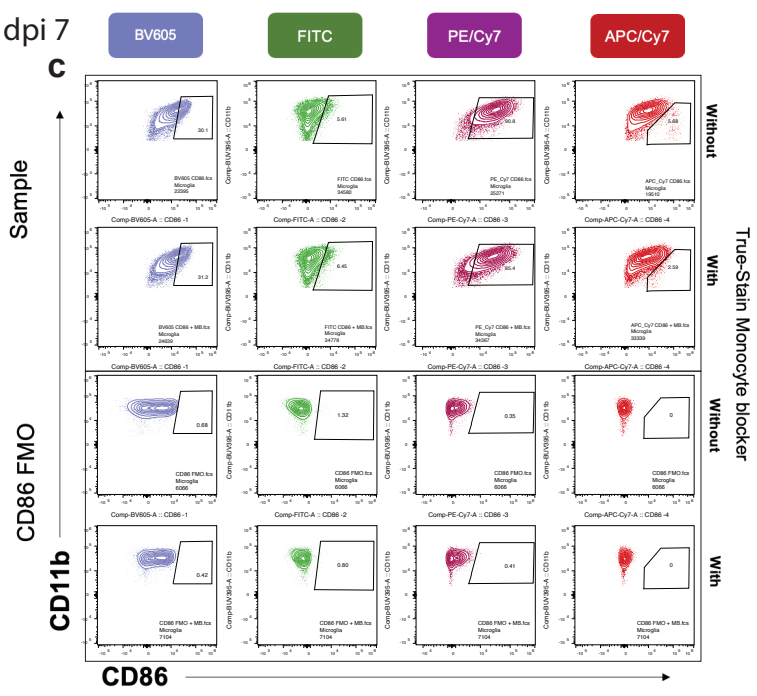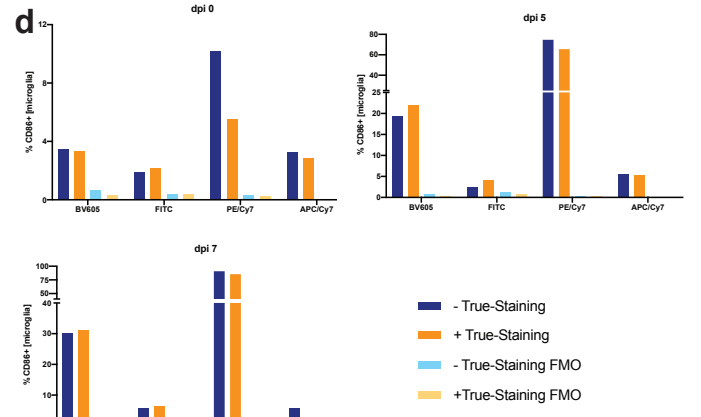

Supplement: Supplementary file 10 — Additional file 10 Brain cells stained with and without cyanine dye blocker and 4 commercially available CD86 conjugates. a-c FACs plots showing murine brain cells from dpi 0 (a), 5 (b) and 7 (c) stained with CD86 conjugated to BV605, FITC, PE/Cy7 and APC/Cy7, with and without True-stain monocyte blockerTM (Biolegend). d Percent of CD86+ microglia from dpi 0, 5 and 7 brains stained with CD86 conjugated to BV605, FITC, PE/Cy7 and APC/Cy7 with and without True-stain monocyte blockerTM. Fluorescence minus one (FMO) stained with and without True-stain monocyte blockerTM (Biolegend) is shown for CD86. [file 12974_2021_2214_MOESM10_ESM.pdf]

**a**

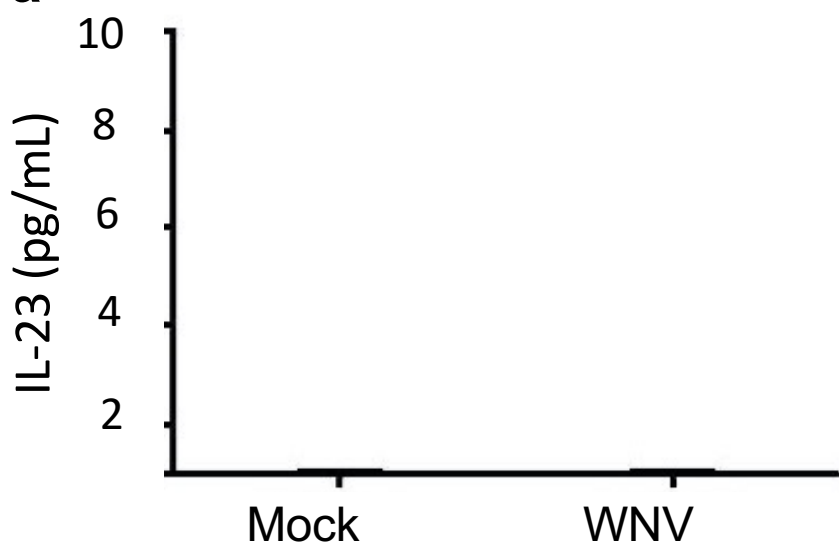

Supplement: Supplementary file 12 — Additional file 12 IL-23 is under the detection threshold in WNV infected brains. Quantity of IL-23 (p19/p40) in mock and WNV-infected mice at dpi 7, as determined using an ELISA. [file 12974_2021_2214_MOESM12_ESM.pdf]
