# Supplementary material for: High-parameter cytometry unmasks microglial cell spatio-temporal response kinetics in severe neuroinflammatory disease
Source: J Neuroinflammation. 2021 Jul 26;18:166. doi: 10.1186/s12974-021-02214-y (PMC8314570; doi:10.1186/s12974-021-02214-y)
Supplement: Supplementary file 7 — Additional file 7 Four microglia phenotypes identified in the homeostatic and infected brain have differential immune profiles. Histograms showing the expression of selected markers on/in P2RY12hiCD86+ (dark purple), P2RY12loCD86- (light green), P2RY12loCD86+ (light purple) and P2RY12hiCD86- (dark green) microglia at dpi 0 (blue) and dpi 7 (orange). [file 12974_2021_2214_MOESM7_ESM.pdf]

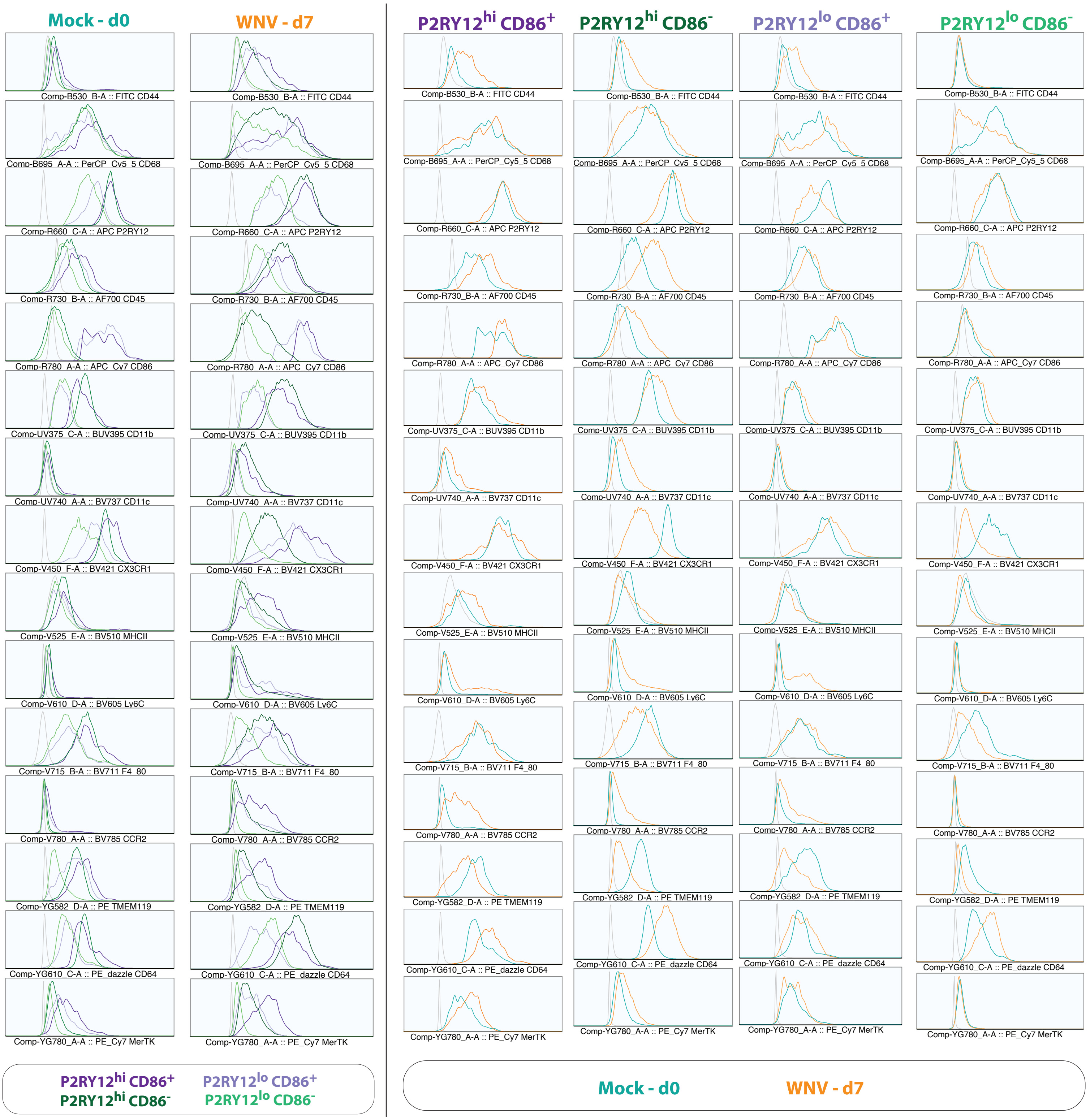
Mock - d0
WNV - d7
P2RY12hi CD86+
P2RY12hi CD86-
P2RY12lo CD86+
P2RY12lo CD86-
Comp-B530_B-A :: FITC CD44
Comp-B695_A-A :: PerCP_Cy5_5 CD68
Comp-R660_C-A :: APC P2RY12
Comp-R730_B-A :: AF700 CD45
Comp-R780_A-A :: APC_Cy7 CD86
Comp-UV375_C-A :: BUV395 CD11b
Comp-UV740_A-A :: BV737 CD11c
Comp-V450_F-A :: BV421 CX3CR1
Comp-V525_E-A :: BV510 MHCII
Comp-V610_D-A :: BV605 Ly6C
Comp-V715_B-A :: BV711 F4_80
Comp-V780_A-A :: BV785 CCR2
Comp-YG582_D-A :: PE TMEM119
Comp-YG610_C-A :: PE_dazzle CD64
Comp-YG780_A-A :: PE_Cy7 MerTK
