# Supplementary material for: High-parameter cytometry unmasks microglial cell spatio-temporal response kinetics in severe neuroinflammatory disease
Source: J Neuroinflammation. 2021 Jul 26;18:166. doi: 10.1186/s12974-021-02214-y (PMC8314570; doi:10.1186/s12974-021-02214-y)
Supplement: Supplementary file 11 — Additional file 11 Temporal immunophenotypic changes in microglial cell populations during WNE. Histograms showing the expression of selected markers on/in P2RY12hiCD86+, P2RY12loCD86-, P2RY12loCD86+ and P2RY12hiCD86- microglia at dpi 0 (purple), 4 (orange), 5 (green), 6 (pink) and 7 (blue). Data is representative of at least two independent experiments. [file 12974_2021_2214_MOESM11_ESM.pdf]

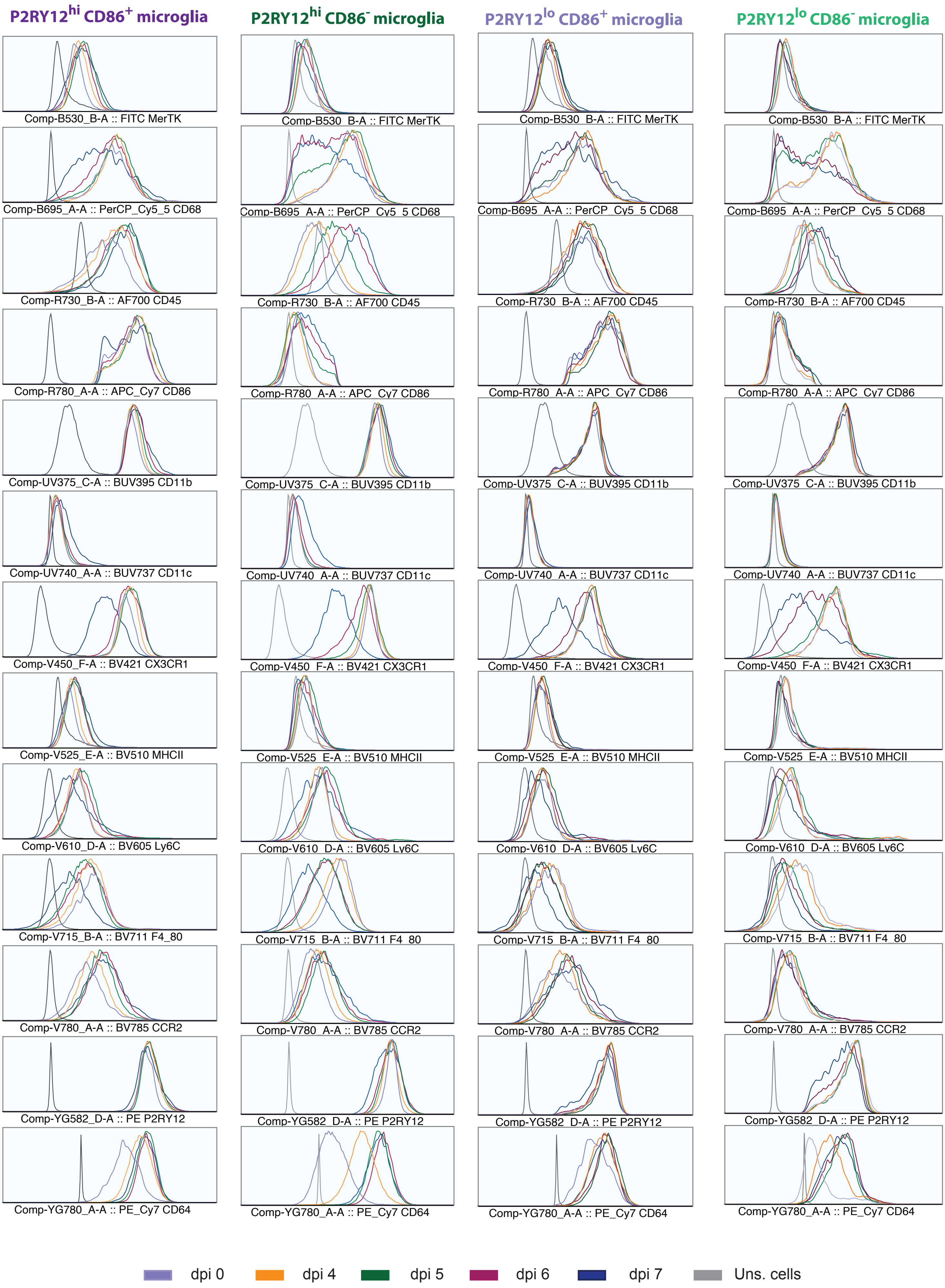
P2RY12hi CD86+ microglia
P2RY12hi CD86- microglia
P2RY12lo CD86+ microglia
P2RY12lo CD86- microglia
Comp-B530_B-A :: FITC MerTK
Comp-B695_A-A :: PerCP_Cy5_5 CD68
Comp-R730_B-A :: AF700 CD45
Comp-R780_A-A :: APC_Cy7 CD86
Comp-UV375_C-A :: BUV395 CD11b
Comp-UV740_A-A :: BUV737 CD11c
Comp-V450_F-A :: BV421 CX3CR1
Comp-V525_E-A :: BV510 MHCII
Comp-V610_D-A :: BV605 Ly6C
Comp-V715_B-A :: BV711 F4_80
Comp-V780_A-A :: BV785 CCR2
Comp-YG582_D-A :: PE P2RY12
Comp-YG780_A-A :: PE_Cy7 CD64
dpi 0
dpi 4
dpi 5
dpi 6
dpi 7
Uns. cells
